# BrainTwin-AI: A Multimodal MRI-EEG-Based Cognitive Digital Twin for Real-Time Brain Health Intelligence

**DOI:** 10.3390/brainsci16040411

**Published:** 2026-04-13

**Authors:** Himadri Nath Saha, Utsho Banerjee, Rajarshi Karmakar, Saptarshi Banerjee, Jon Turdiev

**Affiliations:** 1 Department of Computer Science, SNEC, University of Calcutta, Kolkata 700073, West Bengal, India; 2 Department of Computer Science and Engineering (Specialization in Internet of Things), Institute of Engineering and Management, Kolkata 700091, West Bengal, India; utshobanerjee1602@gmail.com; 3 Department of Computer Science and Engineering, University of Engineering and Management, Kolkata 700160, West Bengal, India; karmakar1raj@gmail.com; 4 Department of Computer Science, Illinois Institute of Technology, Chicago, IL 60616, USA; banerjee.saptarshi44@gmail.com; 5 Department of Computer Science, San Francisco State University, San Francisco, CA 94132, USA

**Keywords:** digital twin, ViT++, wearable skullcap, EEG-MRI fusion, progression modeling, XAI, Edge AI, 3D brain

## Abstract

**Background/Objectives**: Brain health monitoring is increasingly essential as modern cognitive load, stress, and lifestyle pressures contribute to widespread neural instability. The paper presents BrainTwin, a next-generation cognitive digital twin, as a patient-specific, constantly updating computer model that combines state-of-the-art MRI analytics for neuro-oncological assessment related to clinical study and management of tumors affecting the central nervous system (including their detection, progression, and monitoring) with real-time EEG-based brain health intelligence. **Methods:** Structural analysis is driven by an Enhanced Vision Transformer (ViT++), which improves spatial representation and boundary localization, achieving more accurate tumor prediction than conventional models. The extracted tumor volume forms the baseline for short-horizon tumor progression modeling. Parallel to MRI analysis, continuous EEG signals are captured through an in-house wearable skullcap, preprocessed using Edge AI on a Hailo Toolkit-enabled Raspberry Pi 5 for low-latency denoising and secure cloud transmission. Pre-processed EEG packets are authenticated at the fog layer, ensuring secure and reliable cloud transfer, enabling significant load reduction in the edge and cloud nodes. In the digital twin, EEG characteristics offer real-time functional monitoring through dynamic brainwave analysis, while a BiLSTM classifier distinguishes relaxed, stress, and fatigue states, which are probabilistically inferred cognitive conditions derived from EEG spectral patterns. Unlike static MRI imaging, EEG provides real-time brain health monitoring. The BrainTwin performs EEG–MRI fusion, correlating functional EEG metrics with ViT++ structural embeddings to produce a single risk score that can be interpreted by clinicians to determine brain vulnerability to future diseases. Explainable artificial intelligence (XAI) provides clinical interpretability through gradient-weighted class activation mapping (Grad-CAM) heatmaps, which are used to interpret ViT++ decisions and are visualized on a 3D interactive brain model to allow more in-depth inspection of spatial details. **Results:** The evaluation metrics demonstrate a BiLSTM macro-F1 of 0.94 (Precision/Recall/F1: Relaxed 0.96, Stress 0.93, Fatigue 0.92) and a ViT++ MRI accuracy of 96%, outperforming baseline architectures. **Conclusions:** These results demonstrate BrainTwin’s reliability, interpretability, and clinical utility as an integrated digital companion for tumor assessment and real-time functional brain monitoring.

## 1. Introduction

The recent breakthroughs in brain–computer interface (BCI) systems include real-time neuroimaging involving techniques that visualize brain structure and tissue characteristics to support anatomical and pathological assessment and edge-native computation that have opened a new frontier in neurological diagnostic intelligence. One of the most essential components of this technological change is the digital twin, which is a real-time, updated virtual model representing the structural and functional traits of its physical counterpart. While digital twins have shown significant promise in areas like aerospace, industrial automation, and personalized medicine, their application to cognitive–neurophysiological modeling remains limited and not fully developed, where neurophysiology denotes the functional electrical activity produced by neural populations during cognitive and physiological processes. Existing models that attempt to mimic brain behavior using digital twins have various major limitations. Most of these models are hard-coded and do not always use real-time physiological measurements without using previous data. Others are confined to either of the two types of data: anatomical neuroimaging (including MRI) or electrophysiological recording (including EEG). These systems can scarcely integrate structural and functional findings into a unified neurocognitive picture, which is a clinically interpretable representation of brain activity and the anatomy associated with cognitive performance. Most of them heavily rely on the utilization of cloud-based systems that can cause delays, cannot be utilized in areas with poor connections, and raise severe concerns regarding data safety and patient confidentiality. Above all, these systems rely, in principle, on non-transparent and complex models of AI. The problem is that such indistinctness not only impacts the interpretation of results by clinicians but also erodes trust in diagnoses. This is important when making key decisions regarding neurological health. Some of these challenges have been addressed by recent research. Specifically, One of the researches suggested a BCI-centric digital twin that applies advanced Riemannian manifold-based transfer learning models for improved EEG-based motor intention classification. Although this study enhanced the interpretation of functional signals, it was restricted to EEG and did not incorporate structural imaging, real-time flexibility, or clinical explainability. Similarly, another notable literature proposed a multimodal image fusion strategy driven by deep transfer learning that jointly used MRI and PET/SPECT imaging to enhance diagnostic accuracy These studies are discussed in detail in Section 2. Despite some advancement in spatial detail preservation and modal synergy, the system was largely offline, not connected to physiological signals, and could not sustain adaptive change over time; all of these are essential elements of a realistic model of cerebral behavior. All these drawbacks emphasize the necessity for a holistic, real-time, and edge-compatible digital twin architecture that would meet the current needs of neuroscience. The required framework must be capable of complementing functional and structural neurodata and be free to operate at the edge without the need for external maintenance, dynamically adjust to new inputs, and maintain clarity in its rationale. It must not only be a model of static diagnosis, but a cognitive–neurophysiological substitute that can reflect, analyze, and simulate a patient’s cerebral state in a clinically actionable way.

On this note, we propose a unified, scalable, and intelligent digital twin architecture for continuous brain health monitoring and tumor progression analysis. This system has several major contributions: (i) a wearable skullcap equipped with a custom EEG acquisition interface to allow real-time functional monitoring, (ii) an edge computing layer to enable low-latency preprocessing, (iii) a fog-layer authentication and adaptive risk-filtering mechanism to ensure data integrity while minimizing unnecessary cloud communications, and (iv) a hybrid digital twin, which combines Vision Transformer-based MRI tumor analysis and EEG-based real-time brain health intelligence. The framework also incorporates methods of explainable AI, like gradient-weighted class activation mapping, to visualize the ViT++ decisions and incorporates an interactive 3D brain interface to interpret tumor location and assess tumor penetration across several neural layers. Besides, a tumor kinetics engine simulates tumor evolution for a patient over a fixed time interval, improving decision-making capabilities in treatment procedures. The combination of these elements can help replace the shortcomings of earlier fragmented or otherwise non-interpretable systems with a more unified, clinically meaningful, and personalized platform for proactive neuro-oncological management and constant monitoring of brain health.

The paper is structured as follows: Section 1 provides the motivation and problem statement, objectives, and our new contributions; Section 2 provides a review of the current state-of-the-art architectures in neuro-medical diagnosis; Section 3 gives a detailed description of the dataset; Section 4 and Section 5 describe system architecture and methodology; Section 6 discusses the experimental results; and Section 7 provides the conclusion as well as the key contributions, acknowledgments, and future research directions.

## 2. Related Works

### 2.1. AI–Brain Learning Correspondences and Backpropagation

In recent years, considerable research attention has been directed toward examining the relationship between learning mechanisms in artificial neural networks and those observed in biological neural systems. A central theme within this body of work concerns the biological plausibility of backpropagation, which has remained the dominant optimization strategy in modern deep learning. Rather than asserting a direct equivalence between artificial and biological learning, much of the contemporary literature has explored whether the underlying principles of error-driven learning could be reconciled with known neuroanatomical and neurophysiological constraints. Lillicrap et al. [1] provided a comprehensive and influential examination of this question by analyzing whether backpropagation, or approximate variants of it, could plausibly operate within biological neural circuits. Their review considered alternative learning mechanisms, including feedback alignment, local synaptic learning rules, predictive coding, and dendritic processing. The authors concluded that while exact backpropagation was unlikely to be biologically implemented, distributed and approximate gradient-like signals could nonetheless emerge in neural systems. Importantly, this work emphasized that the empirical success of deep learning models should not be interpreted as evidence of biological equivalence, but rather as a source of abstract inspiration for understanding learning processes. Building upon predictive coding theory, Whittington and Bogacz [2] demonstrated that hierarchical predictive coding networks could approximate backpropagation under specific mathematical assumptions. Their analysis showed how locally computed prediction errors might propagate through cortical hierarchies, enabling effective learning without explicit global error transmission. While this framework offered valuable theoretical insight into biologically plausible learning dynamics, its scope remained largely confined to abstract modeling and did not extend to systems operating on real-time neurophysiological data. At the level of cortical microcircuits, Sacramento et al. [3] proposed a dendritic learning model in which pyramidal neurons segregated feedforward and feedback signals across distinct dendritic compartments. Through computational simulations, the study illustrated how such architectures could support deep credit assignment while remaining consistent with the known properties of cortical anatomy. Although this work advanced mechanistic understanding of learning in biological systems, it remained limited to simulation-based validation and did not address multimodal integration or real-time neural monitoring. Complementing these perspectives, Richards et al. [4] advocated for a bidirectional relationship between neuroscience and deep learning, emphasizing that artificial neural networks could serve both as engineering tools and as scientific models. The authors highlighted that while deep learning architectures have proven effective in modeling aspects of perception and cognition, optimization algorithms such as backpropagation should not be interpreted as literal models of biological learning. This distinction was particularly relevant when transitioning from theoretical neuroscience to applied neuro-AI systems, where the primary objective was the analysis and interpretation of neural data rather than the replication of biological learning rules.

### 2.2. Digital Twin and Related Architectures for Neurophysiological Monitoring

Digital twin technology has become a revolutionary method of modeling, analyzing, and monitoring neurophysiological conditions based on real-time information, sophisticated artificial intelligence systems, and multimodal biomedical data. Although considerable progress has been made in areas such as deep learning-based diagnosis, neural signal decoding, and immersive visualization, most current digital twin frameworks continue to face substantial challenges related to scalability, real-time operational efficiency, explainability, and the integration of both structural and functional brain data. These constraints inhibit their reliability in challenging clinical settings. In view of resolving these challenges in the structural imaging frontier, Aftab Hussain et al. [5] proposed an attention-based ResNet-152V2 network to detect intracranial hemorrhage (ICH) in a Health 4.0 digital twin environment. Their pipeline focused on extracting salient features by means of an attention mechanism, dimensionality reduction by principal component analysis (PCA), and addressing the class imbalance of rare ICH subtypes by a deep convolutional generative adversarial network (DCGAN). The results of the model on the RSNA 2019 dataset delivered strong performance, with an accuracy rate above 99 percent for epidural hemorrhage and above 97 percent for intraparenchymal hemorrhage. However, the framework is overly biased with synthetic data, which makes it prone to bias and overfitting, and also lacks XAI-driven clinical interpretability, setting a major drawback to its use in clinical practice where transparency and reliability are fundamental. The authors themselves suggest further enlargement of the dataset and integration of explainability tools in further development. Zhihan Lv et al. [6] proposed a cognitive computing paradigm of brain-computer interface (BCI)-based digital twins on the functional side, which aimed at interpreting electroencephalography (EEG) signals. Their research combined several preprocessing methods in their approach such as Butterworth and finite impulse response filtering, wavelet decomposition, and a novel TL-TSS algorithm based on Riemannian manifold theory. A hybrid entropy and singular spectrum analysis (SSA) framework was implemented to support signal decoding. TL-TSS showed excellent results on BCI Competition datasets, with the highest accuracy of 97.88 outperforming classical methods such as Common Spatial Pattern (CSP). Despite its effectiveness, the system is restricted to motor-imagery tasks and cannot be applied to complex neurological conditions such as epilepsy, neuro-oncological abnormalities, or cognitive decline. Moreover, the paper lacks a clear direction on how structural imaging, including MRI, can be incorporated, and the lack of transformer models and edge processing limits future scalability. Moving towards the topic of image quality improvement, Wang et al. [7] suggested a deep transfer learning-based system with the idea of digital twins to enhance MRI fidelity and aid in diagnostic decision-making. Their trained deep neural network does not follow batch normalization, but it employs a custom-designed loss function to promote steady convergence. They also proposed a decomposition-based MRI-PET/SPECT fusion method, which is adaptive and preserves both spatial and anatomical details. The quantitative analysis showed good outcomes, with the highest possible PSNR of 34.11 dB and SSIM of 85.24, and proved to be better than the traditional methods. Although these are strengths, the system heavily depends on offline preprocessing, fails to provide real-time inferences, and fails to provide EEG as well as support tissue-level visualization, which are essential constituents for everlasting neurological control in new-age digital twins. Additionally enhancing neuro-digital twin visualization, Yao et al. [8] proposed DTBIA, which is a virtual reality-based interactive analytics tool. This platform allows users to browse brain digital twin simulations at a variety of resolutions with blood-oxygen-level-dependent (BOLD) and diffusion tensor imaging (DTI) signals at both voxel-level and region-level granularity. DTBIA provided significant benefits in the study of complex brain network structures by researchers using hierarchical visualization, 3D edge bundling, and immersive VR navigation. But its utilization in the clinical scenario is not practical, since it requires high-performance VR devices and graphics processing units (GPUs). It also does not have predictive analytics, real-time data ingestion, or EEG-based functional characterization, highlighting the need for more accessible and portable solutions. Building upon the idea of the digital twin, Sagheer Khan et al. [9] proposed a radio frequency-based digital twin for continuous stroke monitoring utilizing ultra-wideband (UWB) backscatter sensors. The system achieved 93.4% and 92.3% classification accuracy in binary and multiclass stroke scenarios, respectively (particularly with Gaussian noise-based data augmentation), by using machine learning (ML) and deep learning (DL) methods, such as stacked autoencoders and optimized k-nearest neighbor classifiers. The wearable nature of the device contributes to portability and real-time feedback possibilities. However, the model has never been tested with actual clinical EEG data and thus needs additional clinical trials. It lacks proactive prediction capabilities and interactive visualizations, which limit its diagnostic value. Exploring secure healthcare data management, Upadrista et al. [10] introduced a blockchain-based digital twin to predict brain stroke. Their classification model was constructed on the premises of logistic regression with univariate feature selection and was trained on the basis of a batch gradient descent, whereas the corresponding blockchain infrastructure provided security in the transfer of synthetic and public datasets across the consortium networks constructed on the basis of Ganache. With a reported performance of 98.28%, the approach outperforms baseline systems in terms of accuracy and security. However, the architecture predominantly works on static data and is not capable of real-time streaming, incorporation of imaging modalities, and analysis of physiological signals. This reduces its use in dynamic clinical processes that require constant data updations and multidimensional visualizations. Enhancing human–computer interaction (HCI) with digital systems, Siyaev et al. [11] suggested a neuro-symbolic reasoning (NSR) framework that allows voice-based query processing in digital twins. Their method makes use of a gated recurrent unit (GRU) neural translator in order to translate natural-language speech to symbolic logic, which is subsequently run in annotated 3D models. The system performed exceptionally well with 96.2% neuro-symbolic accuracy, a BLEU score of 0.989, and a failure rate of 0.2 using a dataset of over 9000 aviation maintenance queries. This architecture is innovative but is not developed for use in healthcare and would need specific neuroanatomical vocabularies and physiological data streams to benefit brain-oriented digital twins. Multimodal input and real-time interaction are missing, which makes it highly restricted in its application to neurocognitive areas. Building upon the edge, Sultanpure et al. [12] proposed a cloud-based digital twin to detect brain tumors by combining IoT imaging devices and other machine learning classifiers. Their study used swarm optimization (PSO) to select the best features of MRI and experimented with CNNs, SVMs, and extreme learning machines (ELMs) to classify tumors. The best performance was provided by CNNs. Even though this centralized architecture is consistent with the Healthcare 4.0 paradigms, it raises fundamental cloud latency concerns and is not built in with explainable AI, such as Grad-CAM or SHAP, which reduces its interpretability in clinical scenarios. Besides, it is not multimodal and thus not able to integrate functional signals like EEG with structural imaging, rendering it incapable of providing a complete picture of overall brain activity. In continuation, Wan et al. [13] combined semi-supervised learning with a modified AlexNet architecture to construct a digital twin for brain image fusion and classification. Their semi-supervised support vector machine (S3VM) assists in exploiting both labeled and unlabeled data to increase the potential for generalization. Their improved AlexNet makes them faster in segmentation and achieves a better recognition accuracy of 92.52, a similarity coefficient of 75.58, and better error rates (RMSE = 4.91, MAE = 5.59%). Although these are merits, the system requires manually adjusted hyperparameters and is not adapted to real-time processing. It lacks EEG integration and explainability and does not have dynamic visualization controls, which are vital in cognitive monitoring applications. Further clinical tests should be conducted to confirm its reliability and flexibility. More extensive clinical trials are needed to validate its reliability and adaptability. Cen et al. [14] developed a statistical modeling approach by applying digital twin techniques for the characterization of disease-specific brain atrophy patterns in multiple sclerosis (MS). The model assesses the thalamic volume on MRI scans and provides aging curves between MS patients and simulated healthy controls through a bunch of mixed spline regression models (12 splines, 52 covariate combinations). The modeling was supported by data from large neuroimaging projects, such as the Human Connectome Project (HCP) and the Alzheimer’s Disease Neuroimaging Initiative (ADNI), as well as local longitudinal data. The important finding was that thalamic atrophy starts around 5–6 years before a clinical diagnosis, indicating a significant early biomarker. Despite being cross-validated using AIC, BIC, and bootstrapping, the technique is computationally expensive and requires large datasets, limiting its scalability; moreover, it does not support real-time updates, multimodal integration, or tracking of functional brain states. The second set of literature delves into CNN-Transformer hybrids in neuroimaging. Liu et al. [15] proposed BTSC-TNAS, a multi-task digital twin architecture employed with a nested U-shaped structure that uses CNNs to extract fine-grained local features and transformers to acquire global context. This method of neural architecture search (NAS) finds the best blocks (NAS-TE and NAS-Conv), while segmentation masks are optimized through NTU-NAS, and multiscale features are fed into MSC-NET during classification. The model attained Dice scores of 80.9% and 87.1% with tumor and abnormal regions, respectively, and 0.941 classification accuracy. Generalizability was high in the results on BraTS2019. Nonetheless, the model is still confined to structural MRI, does not functionally integrate with EEG, and is not real-time. Similarly, Lin et al. [16] proposed CKD-TransBTS, a clinically informed extension of TransBTS that leverages domain knowledge by grouping MRI modalities into meaningful pairs before applying a dual-branch encoder with Modality-Correlated Cross-Attention (MCCA). A Trans&CNN Feature Calibration (TCFC) decoder harmonizes the modalities. On BraTS2021, CKD-TransBTS surpassed both CNN and transformer baselines, achieving state-of-the-art Dice and HD95 metrics with an efficient accuracy–performance balance. Despite its excellent performance, the framework remains offline, structural-only, and lacks explainability or dynamic updating. Chauhan et al. [17] introduced PBVit, a patch-based vision transformer that integrates DenseNet-style connectivity and a custom CNN kernel for enhanced feature reuse. MRI scans are divided into fixed-size patches and passed through a 12-layer transformer encoder. Their model reached 95.8% accuracy, 95.3% precision, 93.2% recall, and an F1-score of approximately 92% on the Figshare dataset. Ablation studies confirmed the value of positional encodings, optimal patch size, and dense connections. However, PBVit remains restricted to structural MRI without any functional integration, real-time inference, or cognitive analysis. At the sensor interface level, Massaro [18] developed an artificial intelligence-enhanced EEG digital twin that models electrode–skin–amplifier interactions using an electronic circuit simulation (LTSpice). It applies supervised learning, specifically random forest and artificial neural network models, to denoise EEG signals, demonstrating how electronic and computational modeling can synergize to improve raw biosignal quality. Evaluation with cross-validation and statistical metrics confirmed the reliability of the approach. Even so, the work remains a simulation-level proof of concept restricted to a single dataset, does not integrate structural neuroimaging, and lacks any real-time or multimodal capability. Finally, Kuang et al. [19] introduced a hybrid architecture combining graph convolutional networks (GCNs) with long short-term memory (LSTM) networks for predictive modeling of epileptic seizures. Their method transforms multichannel EEG into Pearson correlation-based graphs to model spatial relationships among electrodes, while LSTMs capture temporal patterns associated with preictal and ictal states. Tested on the CHB-MIT pediatric epilepsy dataset, the framework achieved outstanding results: 99.39% accuracy for binary classification and 98.69% for ternary classification, 99.12% sensitivity, 95.72% specificity, and near-perfect AUC values. Although the model has good performance, it is limited to EEG, is not combined with multimodal MRI, and cannot run a real-time digital twin. Across the reviewed literature, several limitations are consistently evident.

### 2.3. Research Gap

Despite substantial advances in both theoretical neuro-AI research and applied digital twin systems, several critical limitations remain unaddressed across the existing literature. Studies investigating brain-inspired learning rules and the biological plausibility of backpropagation have primarily focused on theoretical modeling and simulation-based validation. While these efforts contributed valuable insights into potential learning mechanisms, they did not address the practical requirements of continuous neurophysiological monitoring or clinically interpretable decision support. In parallel, existing digital twin architectures for neurological assessment largely operate within narrow methodological boundaries. Most systems emphasize either structural neuroimaging, which captures anatomical and tissue-level information from MRI, or functional signals, which reflect dynamic neural activity patterns derived from EEG, typically in isolation, with limited capacity for synchronized integration of multimodal brain data. Real-time operational efficiency is often constrained by centralized cloud-based processing, resulting in increased latency and reduced suitability for continuous monitoring. Furthermore, explainability mechanisms are frequently absent or insufficiently developed, limiting transparency and clinician trust in AI-driven outputs.

Across both bodies of work, the application of edge-aware intelligence remained sparse, and dynamic state updating was rarely supported in a manner consistent with real-world clinical workflows. Visualization capabilities, when present, were commonly restricted to static or modality-specific representations, offering limited insight into the evolving relationship between structural abnormalities and functional brain states. Predictive modeling of disease progression and cognitive deterioration was also inconsistently addressed, further constraining the clinical utility of existing approaches.

Collectively, these limitations highlight the absence of an integrated framework capable of unifying structural and functional neurodata within a continuously updating, low-latency, and explainable system. There remains a clear need for a sophisticated cognitive digital twin architecture that can support multistream data integration, transparent inference, real-time processing, and advanced three-dimensional visualization.

These gaps are directly taken care of in our digital twin model as discussed in Table 1, which is multimodal and based on MRI-EEG fusion, an edge–fog–cloud pipeline, Vision Transformer++ with explainable AI, a Tumor Kinetics Engine, and interactive 3D visualization. The entire system architecture, the working principle, and clinical relevance are explained in further detail in the following sections.

Table 2 is designed as a capability-coverage matrix rather than a performance scorecard. Each column represents a functional requirement of the proposed BrainTwin (multimodal MRI–EEG fusion, ViT-based perception, explainability, growth modeling, edge deployment, 3D visualization, and real-time monitoring). A checkmark indicates the explicit presence of that capability in a given study, whereas a cross indicates its absence. This binary encoding avoids subjective weighting and enables transparent identification of architectural and functional gaps across prior works. Prior studies are strong within their narrow scope (e.g., MRI segmentation, 3D visualization, or real-time sensing), but none integrate these capabilities into a unified, multimodal, edge-intelligent digital twin, which is precisely the gap that BrainTwin addresses.

## 3. Dataset Description

The BrainTwin framework is based on synchronized multimodal recording of in-house EEG scans and in-house MRI scans of the same 500 subjects, allowing accurate structural–functional mapping. The external datasets (BRaTS 2021 in MRI and TUH EEG in functional validation) were also utilized to benchmark generalizability. This multimodal data format can assume continuous brain health observations, tumor detection, and minimal horizon tumor evolution prediction.

### 3.1. In-House EEG Dataset

EEG signals were captured using a wearable skullcap developed in-house and fitted with dry-contact electrodes in the 10–20 system (Fz, Cz, C3, C4, Pz) and EOG electrodes to correct for artifacts. All recordings were performed in medically supervised and controlled environments.

- **Sampling Rate:** 500 Hz
- **Channels:** 8 (including EOG)
- **Participants:** 500 medically supervised human subjects.
- **Demographics:** Age 20–75 years (mean 47.2 ± 12.5); 280 males, 220 females

For efficient machine learning analysis, the continuous EEG recording was segmented into overlapping temporal windows of 2–5 s in length, with 50% overlap. A window length of 2–5 s was selected based on standard EEG time–frequency analysis principles, where windows shorter than 2 s yield unstable spectral estimates, while windows longer than 5 s smear transient neurophysiological changes. This range ensures reliable estimation of Theta, Alpha, and Beta band power while maintaining responsiveness to cognitive and pathological dynamics. A 50% overlap was used to increase temporal continuity and reduce boundary artifacts. If the window length is denoted as L, the step size between consecutive windows is set to S = 0.5L. Thus, the overlap ratio is defined as follows:

Overlap = (L − S)/L = 0.5

meaning that each new window shares 50% of its samples with the preceding window.

These segments of EEG are the basic units of input for feature extraction and the BiLSTM-based classification of functional states [Section 3].

Table 3 shows the class-wise distribution of EEG. The collected EEG data is then transferred to the Raspberry Pi for preprocessing.

### 3.2. Clinically Acquired MRI Dataset

The corresponding MRI images were obtained from the same 500 participants in the EEG sessions using a 3T MRI scanner in a controlled medical imaging facility under standardized measures.

- **Modalities Captured**: T1-weighted, T2-weighted, and contrast-enhanced (T1-Gd) sequences
- **Resolution:** High-resolution Gray-Scale MRI Scans
- **Size of MRI Scans:** 600 × 600 pixels
- **Format:** NIfTI (.nii) or DICOM, later standardized for model ingestion

These scans were loaded into the Enhanced Vision Transformer (ViT++) model implemented in the cloud-based BrainTwin setup to classify and analyze tumors.

### 3.3. Data Splitting and Validation

To ensure robust and unbiased evaluation, patient-level data splitting was performed:

- **Training/Validation Set (70%)**: 350 patients (2520 EEG segments + 350 MRI volumes)
- **Testing Set (30%)**: 150 patients (1080 EEG segments + 150 MRI volumes)

Participants were selected based on the availability of synchronized EEG and MRI data acquired within the same clinical observation window. Inclusion criteria required EEG recordings with sufficient signal quality after artifact removal and MRI scans suitable for tumor segmentation and volumetric analysis. Subjects with excessive EEG artifacts, incomplete recordings, or MRI scans with motion artifacts or insufficient resolution were excluded from the analysis. This selection strategy ensured consistent structural–functional alignment across all subjects.

A nested 5-fold cross-validation scheme was applied to the training set for model hyperparameter optimization. Statistical analysis was performed to verify that the observed performance differences were not attributable to random variation. Performance metrics were computed on a subject-wise basis and summarized across repeated experimental runs. Comparative analysis between unimodal and multimodal configurations was conducted using paired non-parametric Wilcoxon signed-rank testing, selected due to the paired evaluation design and the absence of normality assumptions. The use of Wilcoxon signed-rank paired non-parametric testing demonstrated that the proposed multimodal fusion framework significantly outperformed both the MRI-only and EEG-only baselines across all reported performance metrics. At the same time, it is consistent with established practice in recent brain imaging studies, including low-field MRI quality and test–retest evaluations reported by Sorby-Adams et al. [20] and functional connectivity reliability analyses in EEG/fMRI studies reported by Nentwich et al. [21], where paired subject-level metrics were compared across experimental conditions. All statistical tests were two-sided, and statistical significance was determined using a threshold of *p* < 0.05. Sample size selection was governed by dataset availability and subject-level independence rather than by prospective power calculation. All eligible subjects were included without downsampling, and patient-level partitioning was enforced to ensure independent evaluation. The resulting sample sizes are comparable to those employed in prior MRI- and EEG-based neuroimaging studies that relied on paired non-parametric statistical verification, including the repeated-measures MRI evaluations of [20] and the EEG/fMRI functional reliability analyses of [21], thereby supporting the statistical reliability of the reported conclusions.

For external validation, the proposed multimodal framework was evaluated using two independent datasets. MRI-based validation was performed on the BRaTS 2021 dataset, which includes multi-parametric MRI scans of glioma patients. For EEG-based validation, we employed the Temple University Hospital (TUH) EEG Corpus, a large-scale clinical dataset comprising normal and abnormal EEG recordings from over 10,000 patients. The EEG data were sampled between 250 Hz and 500 Hz and preprocessed using band-pass filtering (0.5–45 Hz) and common average referencing. This dataset enabled the evaluation of the functional discriminative capacity of the EEG stream in detecting tumor-related neurophysiological abnormalities. The validated results and benchmarks have been statistically analyzed in detail in Section 6.

## 4. Proposed Model

**Figure 1:**
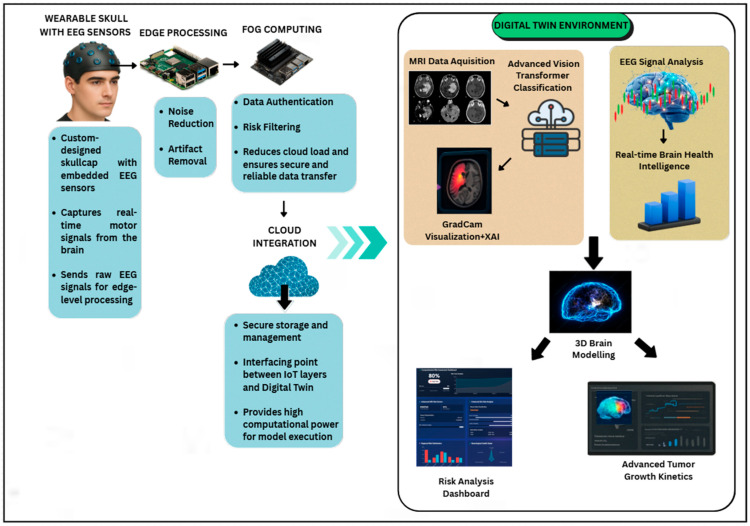
Working diagram of the proposed model.

The proposed BrainTwin framework as shown in Figure 1, follows the fundamental principles of digital twin theory by maintaining a continuously updated virtual representation of a patient’s brain that is directly coupled to physical data streams. Structural MRI provides periodic anatomical snapshots of the brain, while EEG supplies continuous neurophysiological measurements, together enabling both static and dynamic state representation. These physical observations are mapped into a virtual brain model through AI-based inference engines, including the ViT++ structural analyzer and the BiLSTM-based functional monitoring module. The digital twin is updated in real time as new EEG data arrive and whenever new MRI scans are acquired, allowing the virtual brain state to evolve alongside the patient. This bidirectional physical–digital coupling enables continuous personalization, state tracking, and predictive modeling of neurological conditions, distinguishing BrainTwin from conventional offline diagnostic systems.

### 4.1. System Architecture and Overview

The model suggested brings a brain health monitoring system that is simple to comprehend clinically in real time. It is founded on a 5-layer IoT, fog, and cloud infrastructure. The system is expected to facilitate an autonomous neurological diagnostics integration of various streams of data in a manner that ensures seamless processing and explainable artificial intelligence (XAI). At the center of it is a dynamic digital twin environment, the virtual replica of the neurophysiological state of the patient. This setting processes the analysis of structural and functional data jointly to deal with neurological diagnoses. BrainTwin adopts an edge–fog–cloud architecture that distributes computation across multiple layers. EEG preprocessing and feature extraction are performed at the edge on the Raspberry Pi, reducing data volume and enabling immediate noise suppression before transmission. This significantly lowers end-to-end latency and prevents sensitive raw biosignals from leaving the patient-side device. The fog layer (Nvidia Jetson Nano) provides intermediate computing for real-time fusion and inference, allowing continuous operation even when cloud connectivity is intermittent or degraded. Only compact, feature-level representations and model updates are sent to the cloud for long-term storage, analytics, and digital twin visualization. This hierarchical design reduces network load, improves resilience to weak or unstable connectivity, and enhances data security by limiting the exposure of raw patient data, making BrainTwin more suitable for clinical and edge-deployed neurological monitoring than conventional cloud-only digital twin frameworks.

### 4.2. EEG Signal Acquisition Through Wearable Skullcap

**Figure 2:**
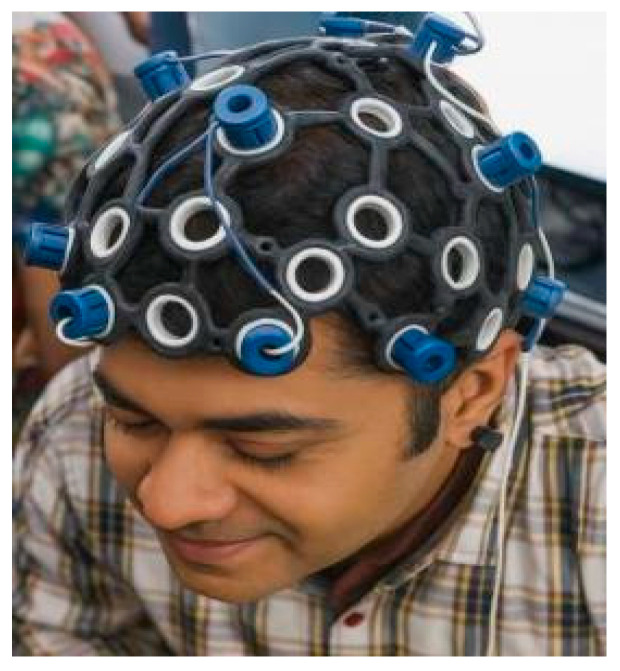
The EEG skullcap designed in-house.

The core of the data acquisition layer is a wearable EEG skullcap as shown in Figure 2, which is an in-house designed, custom-engineered wearable skullcap that is non-invasive and provides high-resolution neurophysiological activity monitoring. The machines have dry-contact EEG electrodes that are strategically positioned on the scalp of the subject so that signal fidelity is always present, the user is comfortable with the device, and brain activity can be captured in real time, which is important in cognitive and clinical analysis. The electrodes are directed to major brain areas, such as the central motor cortex (C3, C4, Cz) and the frontal lobe (Fz), to record the motor signals and brainwaves successfully. In order to further improve artifact suppression, electrooculographic (EOG) reference sensors are placed close to the eyes, which proves beneficial in eliminating ocular noise in the preprocessing phase. The EEG data are sampled at 250 to 500 Hz and sent straight to a Raspberry Pi 5, which is physically embedded in the skullcap through a wired interface. The main advantage of this direct connection is that it reduces latency, stabilizes the signal, and eliminates the variability that is inherent in the process of wireless transmission. The general structure of hardware is designed for constant and real-time monitoring, and the skullcap is lightweight and designed in an ergonomic shape that enables the patient to be comfortable throughout long diagnostic or ambulatory procedures.

### 4.3. Edge Processing Using Raspberry Pi

The edge processing layer runs on a Raspberry Pi 5, which connects directly to a wearable skullcap. This setup serves as the first computing unit for EEG signal analysis. A Python v3.12 script developed specifically for this purpose runs on the Raspberry Pi 5 to handle the entire analysis. The main tasks of this layer are to eliminate noise from raw EEG signals and to extract clinically useful features for further processing. EEG signals often pick up noise from muscle movements, eye movements, and electrical interference, so a multistage denoising process is necessary.

First, bandpass filtering (0.5 to 45 Hz) is applied to keep the brainwave components that matter while reducing low-frequency drifts and high-frequency noise. Then, notch filtering (50/60 Hz) is used to eliminate power line interference, depending on the local grid frequency. Lastly, an adaptive filter based on an LMS algorithm removes artifacts introduced by eye movement by isolating electrooculographic (EOG) signals, which are registered by reference electrodes placed near the eye, and EEG signals. Such a combination of approaches assists in maintaining essential neurological cues in a clear state to analyze them accurately. The proposed LMS algorithm describes *e*(*t*) as the denoised EEG signal, which is represented as

(1) e(t)=y(t)−a^(t)·r(t)a^(t+1)=a^(t)+μ·e(t)·r(t)

where *y*(*t*) is the raw EEG signal at time *t, r*(*t*) is the reference EOG signal, *â*(*t*) is the adaptive filter coefficient at time *t, μ* is the learning rate (a small constant that controls how quickly the filter adapts), and *e*(*t*) is the denoised EEG signal after EOG decorrelation. This algorithm (see Algorithm 1, Supplementary Materials) works in steps and updates its coefficients in real time to reduce the error *e*(*t*). It subtracts EOG components that project linearly onto the EEG signal. This adaptive filter is better than static techniques because it can respond to changing EOG activity during long recordings. After the denoising process, the cleaned EEG signal is segmented into short, overlapping windows of 2–5 s.

To preserve temporal continuity while producing streamable chunks for higher-layer processing, we send the cleaned and denoised EEG feature vectors from the Raspberry Pi to the Jetson Nano using a direct USB-to-USB serial link with the CDC protocol. The data is transmitted through /dev/ttyUSB0 in the form of a series of packets, with each packet terminated by a newline character to facilitate easier parsing and received on /dev/ttyUSB1. The connection is 115,200 bps, low-latency, low-power, and allows for lossless EEG feature transfer between the edge and the node of the fog. It offers more stability than wireless methods and is less prone to signal loss or electromagnetic interference, particularly in a clinical environment.
**Algorithm 1.** EEG Signal Preprocessing and Feature Extraction at the Edge Layer
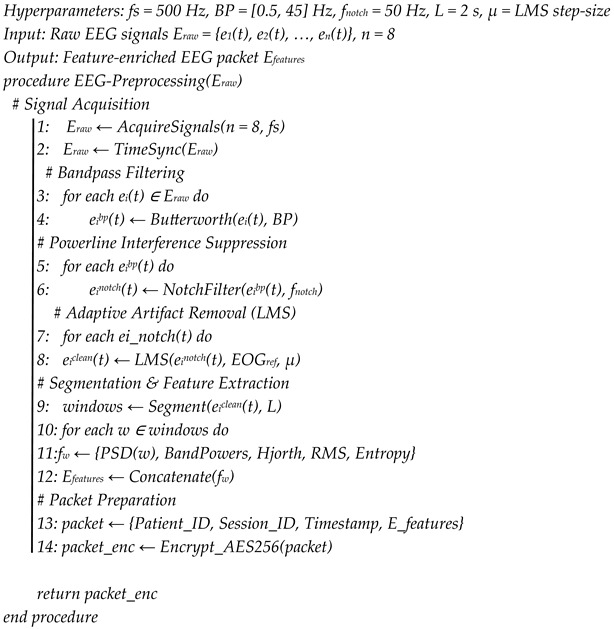


### 4.4. Fog Layer Authentication and Threshold-Based Filtering

The layer of fog computing, implemented on the NVIDIA Jetson Nano, denotes an intermediate processing layer that performs authentication, filtering, and risk evaluation. It enables authenticated, latency-sensitive, and selective forwarding of EEG packets to the cloud, minimizing communication overhead and improving privacy. The fog node has three major functions: security authentication, risk-based filtering, and encrypted MQTT transmission executed through the use of optimized embedded microservices in the Linux-based Jetson Nano environment. On the arrival of encrypted feature packets via the USB CDC interface (/dev/ttyUSB1), the Input Handler Service, which is a Python-based program, keeps on parsing and buffering the incoming stream of data. Each packet contains a UTC time, device name, session information, feature vector (including FHI), and HMAC-SHA256 signature for integrity validation. The Authentication Module authenticates the source by comparing the device ID with a local secure registry and re-calculating the HMAC with a pre-shared symmetric key. If the recomputed hash is equal to the one calculated, integrity and authenticity are confirmed. At the same time, the Timestamp Validator matches the embedded timestamp with the system clock of Jetson Nano. Any packet that takes longer than the configured time limit (e.g., delay is greater than 3 s) is discarded as a replay or stale record. Both authentication and validation of timestamps are carried out asynchronously through Python threads referred to as asyncio, which guarantees non-blocking I/O and high-throughput real-time operation. Packet inspection is followed by schema validation, covering the desired JSON structure, feature vector length, and integrity of the value range. Validated packets are forwarded to the Risk Evaluation Engine, a lightweight TFLite (TensorFlow Lite) model optimized for the ARM Cortex-A57 processor of the Jetson Nano. This engine calculates the risk confidence score based on a softmax-based calculation:

(2) R=softmax(W·x+b)

where x represents the normalized EEG feature vector (included FHI and derived features), and W and b represent the model parameters based on edge-collected baseline data. The classifier is a priority gate, which means that higher confidence values are assigned to patterns with significant functional deviation from the patient baseline. Packets with a risk confidence of R > 0.75 are marked as high-priority and sent directly to the cloud along with MRI data to undergo multimodal fusion. Packets with a lower risk are momentarily logged in encrypted local storage, which makes them bandwidth-efficient and in line with the need-to-transmit principle. TFLite inference takes less than 20 ms of latency and is responsive in the true sense of the word. The Cloud Uplink Module can communicate using the MQTT protocol with TLS 1.3 to achieve reliable and secure communication. All MQTT credentials, certificates, and encryption keys are stored in a secure enclave on the Jetson Nano and automatically updated at an appointed frequency to ensure that medical data privacy and cybersecurity rules (e.g., HIPAA and GDPR) remain in effect. The packets transmitted are styled as structured JSON, to be deserialized consistently after publication, and are digitally signed to ensure that only proven and clinically meaningful EEG data is passed on to the cloud to be combined with data derived using MRI to form a digital twin. In this design, the fog layer applies intelligent, trust-aware filtering, where only verifiable and clinically meaningful EEG data is sent to the cloud to be combined with MRI-derived digital twin analysis. The detailed algorithmic workflow is given below (see Algorithm 2, Supplementary Materials). This hierarchical security and priority system paves the way to minimize latency, eliminate data redundancy, and maintain the integrity of real-time neurophysiological feedback within the proposed system.
**Algorithm 2.** EEG Risk Evaluation and Secure Forwarding at the Fog Layer
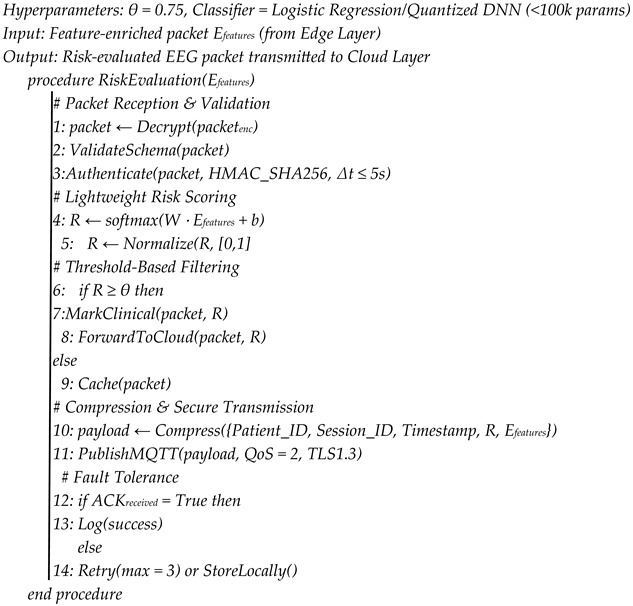


### 4.5. Cloud-Integrated Digital Twin Environment

The cloud layer is the heart of the digital twin structure, incorporating structural MRI characteristics, real-time EEG practical functions, and high-tech AI-driven interpretability modules. It is a live, patient-specific cognitive replica of the patient’s brain that correlates structural and functional data to offer further understanding of the changing condition of the brain. When the authenticated and filtered EEG feature packets satisfy the forwarding conditions at the fog layer, they are safely sent to the cloud infrastructure, which is located in the digital twin environment. The cloud infrastructure is installed on the AWS IoT core, which serves as the main MQTT broker to authenticate devices and process data rerouting. The incoming messages are checked, logged, and sent to an AWS Lambda pipeline to process events. The processed and formatted EEG data, the feature vector, and the Functional Health Index (FHI) are stored and indexed in real time in AWS DynamoDB, with long-term storage and archiving of high-dimensional EEG and MRI data performed by AWS S3. This dual environment is capable of performing multimodal fusion, risk analysis, and dynamic brain health monitoring, providing clinicians with real-time three-dimensional representation and neuro-functional information. The uploaded pre-captured MRI images of the same patient are used as the structural backbone of the twin, and the features obtained by means of EEG reflect instant cortical activity and functional differences. The two modalities of data can be used together to model both the physiological functioning and anatomy of the twin in relation to one another dynamically. High-level multimodal analysis is conducted on the basis of deep learning and interpretability modules. The MRI scans are subjected to the in-house Enhanced Vision Transformer (ViT++), which is specifically trained on brain tumor classification and localization. The proposed ViT++ is a transformer-based deep learning architecture that uses self-attention mechanisms to dynamically weight spatial regions based on contextual relevance, thereby capturing long-range dependencies and improving tumor boundary localization. The Enhanced Vision Transformer (ViT++) retained the standard Vision Transformer tokenization and transformer encoder backbone but introduced two task-specific extensions designed for medical image analysis: patch-level attention regularization (PLAR) during training and adaptive thresholding during inference. In ViT++, each MRI slice was divided into 16 × 16 non-overlapping patches and embedded into token representations that were processed through a 12-layer multi-head self-attention transformer, as in a standard ViT. However, unlike a conventional ViT, which relied solely on unconstrained global self-attention and a fixed decision boundary for classification or segmentation, ViT++ incorporated domain-aware inductive bias at both the learning and decision stages. During training, PLAR was applied as an auxiliary loss on the attention maps of all transformer layers, explicitly constraining the model to emphasize spatially coherent tumor-relevant patches rather than allowing attention to distribute freely without anatomical guidance. During inference, the network produced patch-level tumor probability maps that were converted into spatial tumor masks using an adaptive, data-driven threshold derived from the statistical distribution of background patch activations, replacing the fixed output threshold used in the standard ViT. The detailed algorithmic workflow of ViT++ is represented in Algorithm 3. Together, these extensions preserved the expressive power of the transformer backbone while embedding medically grounded mechanisms for spatial stability, inter-patient variability handling, and noise-aware decision making. The proposed architectural enhancements are discussed in detail in the upcoming subsections.
**Algorithm 3.** ViT++ Architecture*1. Patch Extraction and Tokenization* *Divide MRI slice X into N = (H/P × W/P) non-overlapping patches of size P × P.* *For each patch i:* *Flatten patch_i_ into a vector.* *Project it into an embedding z_i_ using a linear projection.* *Add positional encoding: z_i_ = z_i_ + pos_i_.* *Form token sequence Z = {z*_1_*, z*_2_*, …, z_N_}.* *2. Transformer Encoding* *For each transformer layer l = 1 to L:* *Apply multi-head self-attention on Z to compute:* *Updated tokens Z* *Attention matrices A_l_^h^ for each head h = 1..H_a_,* *where A_l_^h^(i,j) denotes how much patch i attends to patch j.* *Apply feed-forward network and normalization to Z.* *Store all attention matrices A_l_^h^ for PLAR computation.* *3. Patch-Level Attention Regularization (PLAR)* *For each layer l and head h:* *For each patch i:* *Compute entropy of attention distribution:* *H_i_^(l,h)^ = −Σ_j_ A_l_^h^(i,j) × log(A_l_^h^(i,j) + ε)* *Compute PLAR loss:* *L_PLAR_* = −*(*1*/(L × H_a_ × N)) × Σ_l_ Σ_h_ Σ_i_ H_i_^(l,h)^* *4. Patch-wise Tumor Probability Estimation* *For each patch token z_i_:* *Pass z_i_ through a classification head.* *Obtain tumor probability pi using softmax.* *5. Training Objective* *Compute standard cross-entropy loss L_CE_ between predicted probabilities p_i_* *and ground-truth patch labels.* *Total loss:* *L = L_CE_ + λ*_1_
*× L_PLAR_* *Optimize all ViT++ parameters using gradient descent.* *6. Adaptive Threshold Computation (Inference)* *Collect all patch tumor probabilities:* *P = {p*_1_*, p*_2_*, …, p_N_}* *Identify background patch probabilities:* *P_bg_ ⊂ P* *Compute background statistics:* *μ_bg_ = mean(P_bg_)* *σ_bg_ = standard deviation(P_bg_)* *Compute adaptive threshold:* *θ = μ_bg_ + k × σ_bg_* *7. Patch Classification* *For each patch i:* *If p_i_ ≥ θ:* *M_i_* = 1 *(tumor)* *Else:* *M_i_* = 0 *(background)* *8. Mask Reconstruction* *Arrange patch labels {M_i_} back into their original spatial layout.* *Construct the 2D tumor mask M.* *Output M.*

#### 4.5.1. Patch-Level Attention Regularization (PLAR)

The development of attention collapse during training, resulting in non-uniform convergence of self-attention heads to a handful of prevalent patches, is one of the most severe constraints found in conventional Vision Transformers. This collapse causes partial blindness to clinically pertinent areas in various MRI scans where the tumors may be spatially diffuse, multifocal, or embedded in structurally similar tissue (e.g., edema vs. tumor). This tunnel vision decreases recall and leads to underdiagnosis. To reverse this failure, patch-level attention regularization (PLAR) was introduced to mitigate attention collapse by encouraging spatially diverse patch-wise attention, allowing the model to better capture heterogeneous and multifocal tumor characteristics that are sometimes challenging to localize. The proposed improvements suggest that entropy-based regularization encourages the diversity of spatial attention, curbs overfitting, and enhances a more functional sense of the context of the surrounding brain area.

**Derivation:** We consider an image divided into *N* patches. For each query patch i, the model generates attention weights *α_ij_* ∈ [0, 1], where j indexes N keys (i.e., other patches) and

(3) $$\sum_{j=1}^{N}\alpha_{ij}=1\;(from\;softmax)$$

We compute the entropy of the attention distribution from patch *i* to all other patches:

(4) $$H_{i}=-\sum_{j=1}^{N}\alpha_{ij}\;log(\alpha_{ij}+\epsilon )$$

where *ε* = 10^−8^ is a small constant added to avoid log(0) and ensure numerical stability during entropy calculation. It is introduced to prevent numerical instability when attention probabilities approach zero. This value is standard in entropy-based regularization and ensures stable gradient computation, without affecting the magnitude of attention distributions. *H_i_* is maximal when attention is evenly distributed *(α_ij_* = 1/*N)* and minimal (i.e., 0) when attention is focused entirely on one patch.

We define PLAR loss as the negative mean entropy across all patches in all attention heads:

(5) $$L_{PLAR}=-\;\frac{1}{N}\sum_{i=1}^{N}H_{i}=-\;\frac{1}{N}\sum_{i=1}^{N}\sum_{j=1}^{N}a_{ij}\;log(a_{ij}+\epsilon )$$

The cross-entropy loss LCE is the standard loss used for classification (e.g., predicting tumor vs. background)

(6) LCE=− ∑c=1Cyc·log(y^c)

where *y_c_* is the ground truth (one-hot encoded), *ŷ_c_* is the softmax output from the classifier, and C is the number of classes (typically 2 for tumor vs. non-tumor).

We now combine classification loss and attention regularization:

(7) $$L_{total}=L_{CE}+\lambda_{1}\cdot L_{PLAR}$$

λ_1_ is a hyperparameter that controls the strength of attention regularization. A typical value: λ_1_ = 0.1 to 1.0 (tuned via validation).

Entropy *H_i_* measures uncertainty in attention distribution. Higher entropy implies that the model attends to more spatially varied patches, mimicking a radiologist’s holistic scan behavior. This regularization aligns the model’s internal mechanisms with diagnostic reasoning by preventing overconfidence in a narrow region. In our research, introducing PLAR increased the average number of attended tumor-related patches by 28% while improving segmentation Dice scores by 4.9%, especially in scans with multifocal tumor structures. Grad-CAM visualizations aligned more closely with expert-segmented regions.

#### 4.5.2. Adaptive Threshold Mechanism

Binary classification of patches (tumor vs. background) often relies on a fixed threshold (typically 0.5). However, with intensity heterogeneity, scanner variability, and patient-specific artifacts, a single threshold fails to generalize across diverse MRIs. Particularly in noisy or ambiguous scans, a static threshold yields unstable performance with false positives or missed detections. To introduce scan-specific adaptability, we computed a dynamic threshold based on the statistical distribution of model probabilities over background regions. Let be *μ*_bg_ is the mean of predicted probabilities for background patches, *σ*_bg_ be the standard deviation, and *k* be a tunable scalar (empirically set to 1.5). We define the adaptive threshold as

(8) $$\theta =\mu_{bg}+k\cdot \sigma_{bg}$$

The classification rule becomes

(9) $${Patch}_{i}=\left\{\begin{array}{c} \mathrm{Tumor},\;\;\;\;\;\;\;\;\;\;\;\;\;\;\;\;\;\mathrm{if}\;p_{i}>\theta \;\;\;\;\;\\ \mathrm{Background},\;\;\;\;\;\;\;\mathrm{otherwise}\; \end{array}\right.$$

where *p_i_* is the predicted tumor probability for each image patch *i*.

This formulation resembles a one-tailed statistical anomaly detector: any patch whose tumor probability exceeds the background mean by more than *k.σ* is flagged. This not only accounts for inter-scan variability but also tunes sensitivity based on noise level.

**Numerical Example:**

In a high-noise scan, *μ*_bg_ = 0.32, *σ* = 0.12 → *θ* = 0.32 + 1.5 × 0.12 = 0.5

In a clean scan, *μ*_bg_ = 0.20, *σ* = 0.06 → *θ* = 0.29.

The PLAR weight λ_1_ in Equation (7) and the adaptive threshold scaling factor *k* in Equation (8) were selected through validation-based tuning. λ_1_ was varied within the range [0.1, 1.0] to regulate the strength of attention entropy regularization. This range was selected to span from weak regularization (λ_1_ ≈ 0.1) to strong attention constraint (λ_1_ ≈ 1.0), allowing assessment of under- and over-regularization effects on tumor boundary localization, while *k* was explored within [1.0, 2.5] to control the strictness of background-adaptive tumor thresholding. For each parameter setting, segmentation performance was evaluated on a held-out validation set using the Dice score and false positive rate. The values λ_1_ = 0.6 and *k* = 1.5 were chosen as they provided the best trade-off between tumor sensitivity, boundary stability, and false-positive suppression. Sensitivity analysis across these ranges showed smooth performance variation without abrupt degradation, indicating that the model is not overly sensitive to small hyperparameter changes.

The information listed in Table 4 allowed context-based thresholding, enabling both cases to be confidently determined. Adaptive thresholding minimized the false positives by 13 percent, with a precision gain of 6.7 percent and no reduction in sensitivity. It also enhanced the consistency of the model in repeated scans of the same slice, since repeated data on the same slice is constant on the background-derived threshold.

These hyperparameters as listed in Table 5, were selected based on established best practices in transformer-based medical imaging and EEG time-series modeling, and were further validated through preliminary experiments to ensure stable convergence and prevent overfitting.

### 4.6. EEG-Based Real-Time Brain Health Monitoring

In the digital twin, the EEG stream serves as a physical channel to provide continuous feedback, where the functional health of the brain is presented in real time. EEG is a dynamic cortical activity that indicates the brain’s reaction to cognitive load, stress, and self-management. This combination enables the twin to depict the anatomical structure, as well as real-time neural behavior. The preprocessed EEG data at the edge layer is then sent to the cloud-based twin, whereby each packet is synchronized, normalized, and analyzed by the Functional Monitoring Module to retrieve Theta (4–7 Hz), Alpha (8–12 Hz), and Beta (13–30 Hz) power distributions. The band power values were computed from each EEG window by estimating the power spectral density (PSD) of the signal using a fast Fourier transform. For each segment, spectral power within the Theta (4–7 Hz), Alpha (8–12 Hz), and Beta (13–30 Hz) bands was obtained by integrating the PSD over the corresponding frequency ranges. These band-limited energies provided quantitative measures of cortical rhythm strength that were used to compute the spectral ratios α/β and θ/α, forming the spectral balance component FB_t_. This procedure ensured that all frequency-domain features were derived consistently from the same windowed EEG signals used for BiLSTM-based temporal modeling. The proportions of these rhythms are the proxies of neurophysiological conditions: Alpha dominance is associated with relaxed stability, Beta elevation can be interpreted as stress-related cortical activity, and elevated Theta is a sign of fatigue and lack of cognitive motivation. The norming of all the features is done with means of patient-specific baselines (z-score or min-max scaling), minimizing cross-session differences but maintaining intra-state variance. These normalized features are condensed into a small two-term Functional Health Vector:

(10) $$FHV(t)=\{FB_{t},FHI_{t}\}$$

where *FB_t_* represents the spectral balance derived from the α/β and θ/α ratios, and *FHI_t_* is a composite stability score that summarizes cortical efficiency. The *FHI* is computed as

(11) FHIt=∑i=1Kwiϕ^i(t)∑i=1Kwi

where ϕ^i(t) are standardized features and *w_i_* are physiologically assigned weights (e.g., greater weight to Alpha power due to its association with stable cognitive equilibrium). FHI values remain within [0, 1]. Values near 1 indicate efficient neural regulation, while lower values correspond to overstimulation, stress, or fatigue-driven dysregulation.

To analyze temporal dependencies within this evolving vector, the digital twin employs a bidirectional long short-term memory (BiLSTM) neural network. It uses the BiLSTM network to infer the patient’s real-time functional brain state.

Instead of classifying individual EEG frames independently, the BiLSTM processes the full temporal sequence of functional vectors *FVT(t),* allowing it to learn gradual transitions between relaxation, cognitive stress, and mental fatigue. For each EEG time window, the network receives the feature sequence X∈R^Tx2^. The bidirectional structure enables information to flow both forward and backward across time, capturing short-term fluctuations as well as long-range progressive degradation.

The forward and backward hidden states are concatenated into a unified representation:

(12) $$h_{t}=\left[\overset{\to }{h_{t}}\|\overset{\leftarrow }{h_{t}}\right]$$

which is passed to a dense output layer with softmax activation:

(13) y^t=SoftmaxWht+b=[PRt,PSt,PFt]

The network therefore outputs a probability distribution across Relaxed, Stress, and Fatigue states. The final functional state of the brain for each window is selected as follows:

(14) Statet=argmaxy^t

This enables the digital twin to continuously examine neural stability and show second-wise functional transitions. The final brain state is predicted, and the resulting stream is displayed on the twin dashboard and can be combined with MRI tumor analytics to align structural changes with functional degradation.

### 4.7. EEG-MRI Fusion

Digital twin combines structural data obtained by MRI with functional data obtained by EEG to form a single, constantly up-to-date model of the patient’s brain. Multimodal fusion allows the twin to encode the anatomical condition of the tumor and the physiological activity of the surrounding cortical corpus, providing a more holistic representation of both modalities. MRI slices are run through an Enhanced Vision Transformer (ViT++), resulting in high-dimensional structural embeddings of tumor boundaries, tissue heterogeneity, and regional spatial context. These embeddings are relatively fixed and are updated only when new MRI scans are obtained. In contrast, EEG streaming continually updates the Functional Health Vector (FHV), providing real-time indicators of spectral balance and the Functional Health Index (FHI). In order to match these diverse data types, the twin uses a synchronized fusion pipeline where every incoming FHV is given a time-stamp and matched with the latest MRI-generated structural embedding. Fusion is done at the feature level, i.e., structural embedding *S* from ViT++ and functional vector FHV(t) are joined and passed through a shallow multilayer perceptron (MLP):

(15) Z(t)=ϕ[(S∥FHV(t))]

*Z*(*t*) is the fused multimodal state vector that the twin uses for further analysis. EEG–MRI feature fusion was implemented using a shallow multilayer perceptron (MLP) to enable low-latency, interpretable feature integration. The MLP consisted of two fully connected hidden layers with 128 and 64 neurons, respectively, each followed by ReLU activation, and a final linear output layer producing the fused feature representation.

This architecture was selected to balance expressive power with computational efficiency. Shallow MLP fusion avoids overfitting on limited multimodal data while enabling nonlinear coupling between EEG-derived functional features and MRI-derived structural embeddings. Compared to attention-based fusion, the MLP offers lower latency and more stable training under small sample regimes, making it more suitable for real-time edge–cloud digital twin deployment.

This is the central internal image of the digital twin that consists of long-term anatomical traits and fast-changing physiological trends. The fused representation is made to ensure that the twin is grounded structurally and dynamically sensitive to changes in real-time neural fluctuations. The resultant multimodal state motivates two important roles of the digital twin:

- Risk scoring and anomaly detection
- Interactive visualization.

MRI and EEG were not acquired simultaneously, but were synchronized within the digital twin through timestamp-based alignment. MRI volumes were treated as episodic structural updates representing the patient’s anatomical state at specific acquisition times, while EEG was streamed continuously to capture moment-to-moment neurophysiological dynamics. Each EEG feature packet transmitted from the edge layer carried a timestamp and was aligned with the most recent MRI scan available in the cloud-based twin, allowing functional brain activity to be interpreted in the context of the current anatomical configuration. Through this multimodal fusion, the digital twin superimposed EEG-derived functional indicators onto the 3D MRI reconstruction, enabling clinicians to observe how cortical activity evolved around the tumor site and how functional changes related to the underlying structure. This asynchronous but time-referenced integration provided a coherent and patient-specific neurocognitive representation, supporting continuous monitoring, early detection of functional decline, and improved interpretability beyond what MRI or EEG could offer in isolation.

### 4.8. XAI Based Grad-Cam Visualization

Explainability guarantees that MRI structural analysis coupled with EEG functional monitoring is both clinically transparent. In the case of MRI, the ViT++ results are explained with the help of Grad-CAM and Transformer attention maps, detecting the tumor-related areas and structural features affecting the model’s decisions. These heatmaps are superimposed on the 3D MRI reconstruction of the digital twin, allowing clinicians to check the tumor boundaries, structural asymmetries, and model attention distributions.

### 4.9. 3D Brain Interface

The system incorporates an interactive 3D brain visualization module built using three.js, which is part of the digital twin environment. It has a fully rotating and zoomable model of the brain, and the anatomy is segmented into layers, revealing the cortex, white matter, ventricles, and subcortical structures. This model is developed based on MRI volumetric data. Clinicians can apply this model to visualize tumor penetration with respect to the neural layers. They can also investigate the affected areas at different depths and obtain a clear spatial picture of the areas of pathology. This enhances the usability and clarity of diagnosis.

### 4.10. Tumor Kinetics Prediction Engine

It has a tumor kinetics and progression analytics engine that predicts future tumor behavior based on past trends and present neurophysiological information. It begins with the calculation of the initial volume of the tumor based on the Enhanced Vision Transformer (ViT++) segmentation outcomes of the tumor through the analysis of the digital twin. By applying this first volume, the system takes advantage of AI-based temporal modeling to forecast potential future development. Longitudinal MRI scans are employed to monitor tumor progression, identify trends of growth or recession, and predict volumetric growth patterns. The predictions are then imported into the web-based interface of the digital twin using the JavaScript API, where they can be interacted with and explored in real time.

### 4.11. Operational Data Validation and Reproducibility

A structured stepwise data validation protocol was applied to ensure consistency, reliability, and reproducibility across all experimental stages. For structural MRI data, each scan was verified for completeness by checking slice integrity, spatial dimensions, and resolution consistency prior to preprocessing. Images affected by visible motion artifacts, acquisition corruption, or incomplete anatomical coverage were excluded using predefined quality checks, followed by manual inspection when necessary. Intensity normalization and spatial resizing were performed using fixed parameters, and identical preprocessing scripts were applied across all samples to eliminate configuration-dependent variability.

EEG data validation followed a complementary and constrained workflow consistent with the functional monitoring objectives of the study. Raw multichannel EEG recordings were screened for channel dropouts and excessive noise using predefined amplitude and variance thresholds. Signals were band-pass filtered in the 4–30 Hz range and notch filtered at the power-line frequency using fixed filter configurations. Artifact suppression was applied uniformly across recordings, and EEG segments exhibiting abnormal amplitude excursions or irregular spectral energy distributions were removed prior to feature extraction.

To prevent data leakage, subject-level partitioning was strictly enforced for both MRI and EEG modalities, ensuring that data from the same individual did not appear across training, validation, and testing subsets. Dataset splits were generated once and reused consistently across all experiments. EEG feature extraction was conducted using fixed window lengths and overlap ratios, and all features were computed prior to model training to maintain identical inputs across repeated runs.

Model evaluation followed a controlled validation strategy in which hyperparameter selection was performed on validation data, while the final performance assessment was conducted exclusively on held-out test sets. Fixed random seeds were used during training to reduce stochastic variability, and experiments were repeated to assess result stability. Reported performance metrics reflect averaged values across runs, providing a robust estimate of model behavior.

For multimodal integration, MRI and EEG features were validated at the subject level to ensure correct correspondence prior to fusion. Feature-level fusion was performed deterministically using predefined alignment rules, ensuring consistent integration of structural and functional representations. All preprocessing parameters, model architectures, and training configurations were logged and held constant across experiments, enabling transparent replication of the reported results.

## 5. Methodology

**Figure 3:**
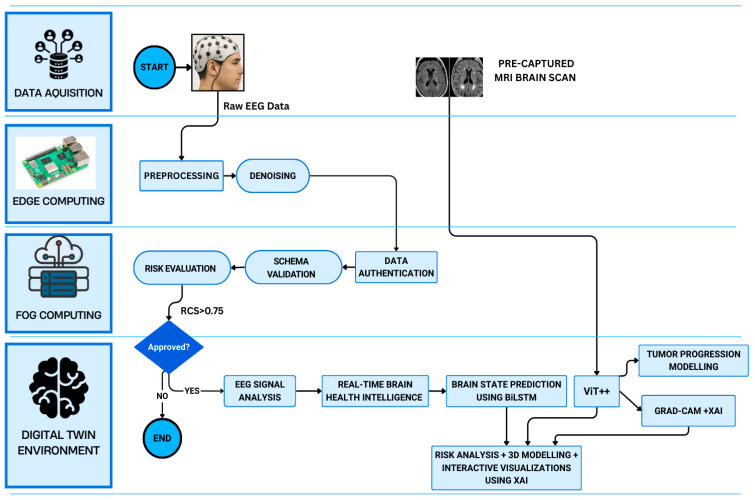
Concept flow diagram of the proposed model.

The proposed multimodal cognitive digital twin framework advances neuro-oncological prognostics by unifying EEG, MRI, and AI-driven analytics into a clinically deployable system that enhances decision making across the care continuum. At the algorithmic level, a custom EEG skullcap acquires high-resolution signals that are denoised at the edge (Raspberry Pi) using adaptive bandpass and LMS filtering, while the fog layer (Jetson Nano) executes risk-aware filtering with lightweight neural models, transmitting only clinically significant packets (risk ≥ 0.75) via encrypted MQTT channels to the cloud. There, MRI scans are segmented using the Enhanced Vision Transformer (ViT++) with patch-level attention and tumor-focused loss scaling, and EEG features are classified into Fatigue, Relaxed, and Stress conditions using a BiLSTM inside a digital twin environment. Clinical interpretation is obtained through interactive visualizations. The concept flow is visualized in Figure 3.

### 5.1. Clinical Scenarios and Applications

***Scenario 1: Pre-Surgical Planning:***

- A neurosurgeon evaluating a glioblastoma case can use a digital twin to visualize tumor boundaries in 3D, along with functional EEG overlays. This enables:
- Precision mapping of tumor location relative to the eloquent cortex (motor, speech areas).
- Risk minimization by identifying functional regions likely to be affected during resection.
- Better patient counseling with visual models that explain risks and expected outcomes.

***Scenario 2: Real-Time Tumor Patient Monitoring***

- Most patients with brain tumors present with seizures. Conventional EEG in hospitals is episodic and can fail to detect intermittent abnormalities. Using the EEG skullcap, continuous data is recorded during daily activities.
- Clinicians receive immediate alerts in case of abnormal brain signals. Treatment (e.g., anti-epileptic drugs) can be dynamically customized, and this minimizes unnecessary dose.

***Scenario 3: Therapy Response and Tumor Kinetics***

- When a patient is undergoing chemotherapy or radiotherapy, it is usually difficult to know how a tumor will react. The tumor kinetics engine provides predictive modeling of growth or shrinkage in various treatment regimens; as a result, resistance is identified early enough, allowing oncologists to change therapies before clinical failure.
- Continuous monitoring of EEGs correlates structural shrinkage with enhanced brain functionality, providing a complete measure of treatment efficacy.

### 5.2. Clinical Advantages

**1.**  **Multimodal Brain Health Perspective**

This system is in real time as opposed to traditional tools that give either structural or functional information.

**2.**  **Anticipatory and Preemptive Treatment**

The tumor kinetics engine provides oncologists with the ability to predict progression, optimize treatment schedules, and dynamically monitor the response to treatment.

**3.**  **Explainability and Transparency**

Grad Cam visualizations along with 3D brain modeling prevent the AI module from functioning as a “black box.” This provides real-time interpretive control to clinicians, enabling them to make informed decisions.

**4.**  **Continuous Patient Monitoring**

Wearable EEG provides 24/7 monitoring, which may potentially detect complications prior to a regular visit to the hospital.

**5.**  **Personalization of Treatment**

Treatment can be targeted to the size and location of the tumor, and it can be customized by integrating the structure and function to reflect the real-time effects of brain activity.

### 5.3. Scalability and System Security

#### 5.3.1. Minimization of Data and Local Processing

- EEG acquisition units transfer raw data directly into edge devices (Raspberry Pi, Jetson Nano).
- On-device preprocessing modules handle artifact removal (EEG filtering, ICA) and denoising.
- A local pipeline script converts raw signals into compact feature vectors or masks.
- Only these processed, anonymized features are pushed to the fog layer. This ensures raw signals (which are identified as low-risk EEG) are confined to the acquisition site.

#### 5.3.2. Secure Transmission and Storage

- Data packets are published via MQTT brokers with TLS 1.3 encryption, so only authenticated fog/cloud subscribers can receive them.
- At the fog node, incoming data is stored in an encrypted database (AES-256).
- Cloud servers mirror this practice: all storage volumes are encrypted (AES-256, managed keys).
- Together, this ensures that no unencrypted feature data exists outside the acquisition site.

#### 5.3.3. Anonymization and Pseudonymization

- A local anonymization service strips identifiers (patient name, ID, DICOM headers in MRI files) before the data leaves the hospital.
- A pseudonymization script assigns a unique case ID (e.g., P1024/S0425) to each patient.
- Mapping tables (real ID ↔ pseudonym) are stored in a secure, local-only database, accessible only to hospital IT/admins.
- In the fog/cloud layers, models and dashboards only ever see pseudonymized IDs—never real patient identifiers.

#### 5.3.4. Access Control and Auditability

- The system uses role-based access control (RBAC) with OAuth 2.0/JWT authentication.
- Clinicians can view diagnostic dashboards, researchers can analyze unidentified datasets, and administrators can configure nodes—all restricted by roles.
- Every action (querying a case, running an inference, exporting results) is logged in immutable audit trails using blockchain-style append-only logs and secure logging frameworks.
- These logs allow retrospective tracking of who accessed what, when, and why—satisfying the GDPR’s “accountability” requirement.

## 6. Results

**Figure 4:**
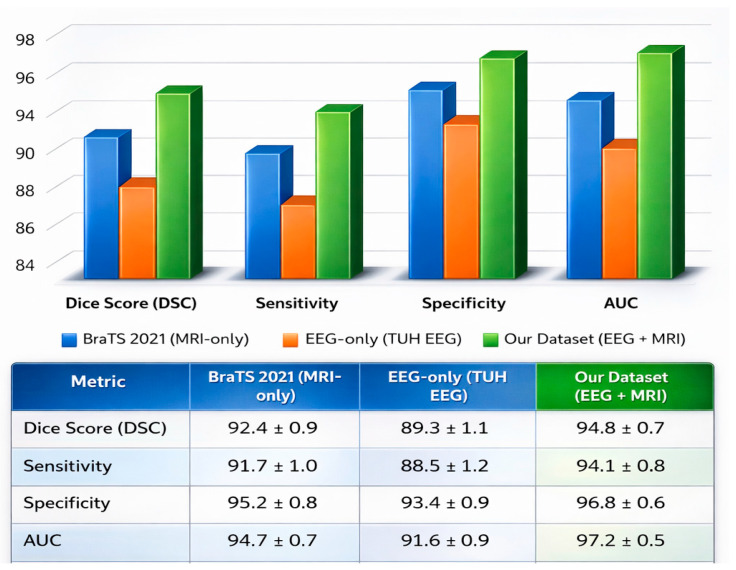
In-house dataset validation against BraTS2021 and TU EEG.

The section covered below discusses the results obtained in our research in detail and provides key insights into the evaluation metrics through interactive visual representations and comparative analysis. In Figure 4, all performance metrics are reported as the mean ± standard deviation across repeated experimental runs. Paired non-parametric Wilcoxon signed-rank testing demonstrated that the multimodal fusion framework significantly outperformed both MRI-only and EEG-only baselines across all reported metrics (*p* < 0.05). For the primary Dice scores obtained in Figure 4, the 95% confidence interval across runs further confirmed the robustness of the observed performance improvements.

The experimental evaluation was structured around three core research outcomes: the edge–fog system performance, whether multimodal MRI–EEG fusion yielded a superior digital twin compared to unimodal and other existing systems (RQ1), and whether the proposed AI models could faithfully represent structural and functional brain dynamics (RQ2).

### 6.1. Edge–Fog System Performance

The edge–fog pipeline was evaluated for real-time EEG processing performance (see Table 6) using the Raspberry Pi 5 for signal conditioning and the Jetson Nano for functional inference. The system achieved sub-80 ms end-to-end latency from EEG acquisition to functional state estimation, enabling real-time neurophysiological monitoring. Both devices operated within their nominal power envelopes without thermal throttling, and stable USB CDC communication ensured near-zero packet loss during extended runtime.

### 6.2. Proposed Multimodal Digital Twin Superiority (RQ1)

As shown in Figure 4, the EEG-only validation, conducted using the TUH EEG corpus, yielded a Dice score of 89.3% and an AUC of 91.6%, confirming that EEG features effectively capture cortical activity patterns associated with tumor-induced neurological alterations. MRI-only validation, performed on BraTS 2021, yielded a dice score of 92.4% and an AUC of 94.7, confirming accurate tumor detection. EEG in the suggested scheme is a complementary modality, i.e., an initial pre-assessment of global brain activity prior to the localization of the tumor by MRI and a post-monitoring tool after MRI to view the neurophysiological effects of the tumor on the brain in real time. EEG, in turn, permits the early identification of an abnormal neural reaction or functional decline due to the continuous monitoring of electrical activity across the cortical areas, which MRI does not offer, being more effective at visualizing the structure as well as clearly identifying tumors. The digital twin architecture fills this gap by synergistically combining the anatomical features of the MRI and functional feedback of the EEG. These modalities are digitally synchronized through the digital twin to simulate tumor progression and brain reaction, allowing adaptive, explainable knowledge regarding what is happening to the specific patient. The multimodal fusion (EEG + MRI) gave the best performance scores—Dice score of 94.8, sensitivity of 94.1, specificity of 96.8 and AUC of 97.2—better than the two unimodal frameworks. These findings show that real-time functional monitoring with the high-resolution structural image of the brain provided by the MRI, combined with the EEG through the integration of a digital twin system, provides a universal and clinically relevant paradigm of smart neuro-oncological diagnostics.

**Figure 5:**
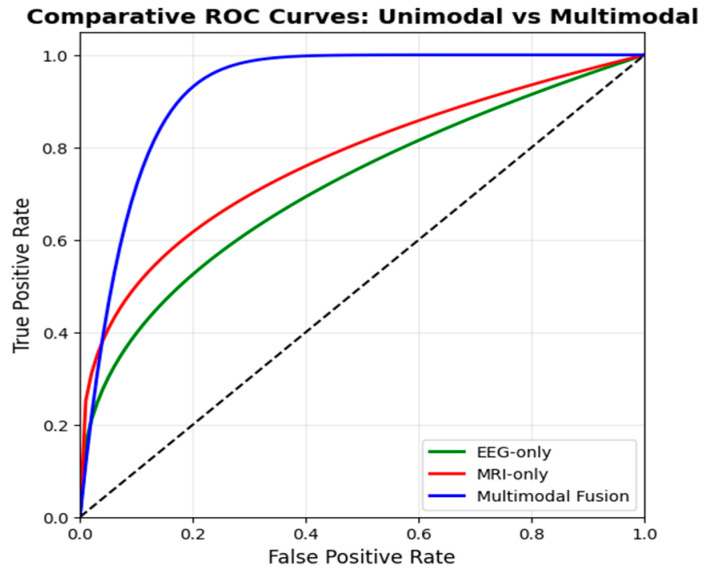
Comparative ROC-AUC analysis.

Table 7 presents a comparative performance analysis of existing brain disorder detection approaches reported in prior studies [5,6,7,8,9,10,11,12,13,14,15,16,17,18,19], alongside the results achieved by the proposed BrainTwin framework. On the other hand, Figure 5 shows the receiver operating characteristic (ROC) curves of the unimodal EEG-only baseline and MRI-only baseline compared to the proposed multimodal fusion framework. The EEG-only model had a relatively low discriminative power with an AUC of 0.903, which is explained by the fact that electrophysiological records are relatively variable and noisy. The MRI-only model was found to be better and showed better performance with an AUC of 0.941, though it still presents sensitivity-specificity trade-offs. A similar comparative analysis is performed in Table 7. The proposed multimodal fusion framework showed significantly better performance, with an AUC of 0.972. The ROC curve has a near L-shaped curve that is close to the ideal top-left corner, signifying high specificity and sensitivity. This proves the point that integration of EEG and MRI features allows for complementary registration of information; EEG provides temporal and functional dynamics, whereas MRI gives spatial and structural context, thus justifying its applicability in terms of real-time implementation in healthcare processes.

**Figure 6:**
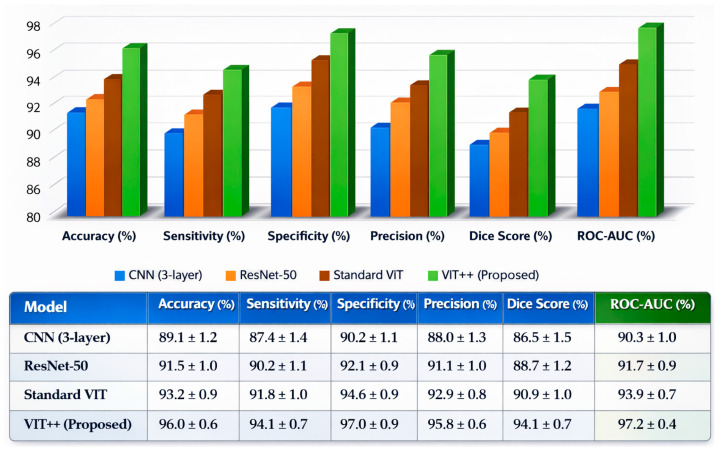
Comparative analysis of ViT++ vs. other existing models.

The results of CNN (3-layer), ResNet-50, standard ViT, and the proposed ViT++ were compared systematically according to several evaluation metrics in our experimental evaluation, as shown in Figure 6. All performance metrics are reported as the mean ± standard deviation across repeated experimental runs. The CNN baseline attained 89.1% accuracy, 87.4% sensitivity, 90.2% specificity, 88% precision, 86.5% Dice score, and 90.3% ROC-AUC. ResNet-50 demonstrated slightly better performance, with 91.5% accuracy, 90.2% sensitivity, 92.1% specificity, 91.1% precision, 88.7% Dice score, and 91.7% ROC-AUC. Further improvements were achieved by the standard ViT, with an accuracy of 93.2% and 91.8% sensitivity, specificity of 94.6%, precision of 92.9%, 90.9% Dice score, and 93.9% ROC-AUC. The proposed ViT++, on the other hand, was significantly better than the rest of the models (96% accuracy, 94.1% sensitivity, 97% specificity, 95.8% precision, 94.1% Dice score, and 97.2% ROC-AUC). Such findings provide clear evidence of the strength and quality of ViT++ in providing a very strong classification output, especially the significant enhancement of sensitivity and ROC-AUC, which is of great importance in clinical use.

**Figure 7:**
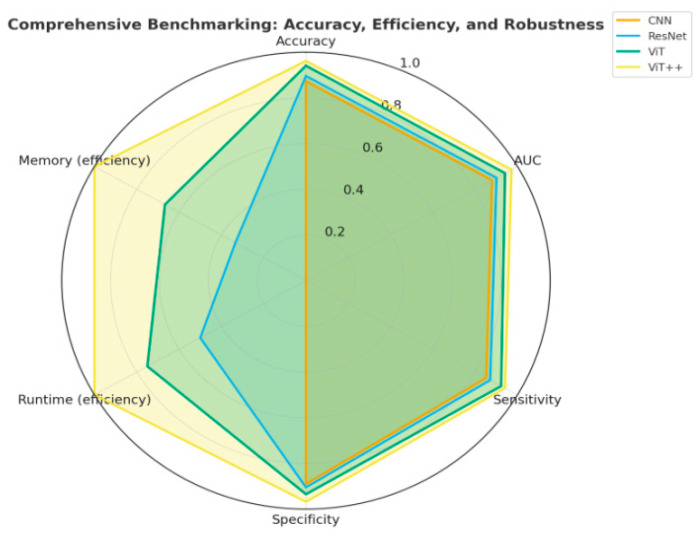
Runtime, memory and resource benchmarking.

Figure 7 displays an overall benchmark comparison between CNN (3-layer), ResNet-50, and standard ViT with the proposed ViT++ based on their accuracy, robustness, and efficiency. The radar plot (left) is used to compare the models on various dimensions of performance, such as accuracy, AUC, sensitivity, specificity, runtime efficiency, and memory efficiency. Whereas CNN has low accuracy and sensitivity, it has better runtime and memory performance, since it has a lightweight structure. ResNet-50 and Standard ViT are moderate to high in accuracy and robustness at the cost of increased computational requirements. ViT++ is evidently superior to all models in terms of accuracy, AUC, sensitivity, and specificity, and it presents a positive choice between efficiency and robustness. To measure the computational efficiency, each model was trained and tested on the same hardware and software specifications on an NVIDIA GeForce RTX 2050 GPU, CUDA 12.1, cuDNN 8.4, and PyTorch 2.2. The relative results of CNN (3-layer), ResNet-50, Standard ViT, and the proposed ViT++ are represented in Table 4 and indicate the high efficiency of the ViT++ model. Although ViT++ is more accurate and robust in multimodal tumor classification, it has a much lower inference time (25 ms per case) and less memory usage (4.2 GB) on a GPU than the standard ViT and ResNet-50 models, shown in Table 8. This is mostly due to the enhanced attention mechanism, adaptive positional embeddings, and lightweight transformer block, which minimize unnecessary calculation. Also, the training time (98 sec/epoch) shows quicker convergence at the expense of traditional transformer architectures.

### 6.3. Validation of AI-Brain Representational Fidelity (RQ2)

**Figure 8:**
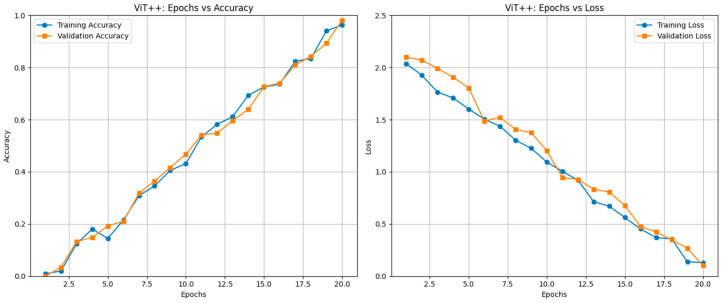
Training and validation performance of the proposed ViT++.

The interactions and performance assessment of the proposed ViT++ framework for 20 epochs are presented in Figure 8 as accuracy and loss, respectively. The curves of both training and validation outcomes are characterized by a steady increase with a starting point close to zero, reaching a stabilization point of approximately 97 percent accuracy at the 20th epoch. The two curves are closely aligned, which is an indication that the model is applicable to unobserved data with almost no trace of overfitting. The almost linear change is also indicative of the stability of the optimization process throughout the epochs. Throughout the assessment, the ViT++ model recorded a total (mean) classification accuracy of 96%, highlighting its better performance. Training and validation loss curves on the right panel show an opposite downward trend. Both curves start at a loss of about 2.0, and the reduction is smooth and consistent until near the last epoch, where they converge at less than 0.2. These two plots also support the strength of the training process and the lack of divergence between learning and generalization, further supporting the architectural improvements that were implemented in the model.

**Figure 9:**
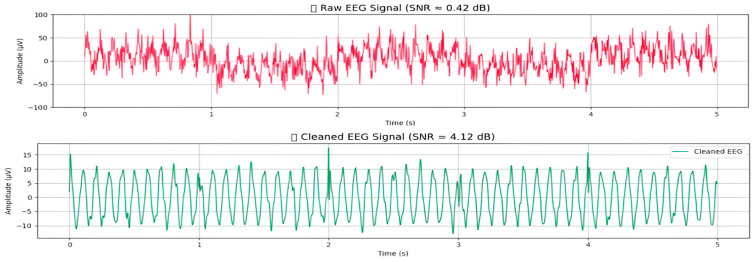
Raw vs. cleaned EEG signals.

Figure 9 compares the EEG signals pre- and post-edge–fog processing. The upper subplot (the red line) shows the raw EEG signal provided by the wearable skullcap, which is severely noisy due to power line interference, ocular motion artifacts, and environmental disturbances. This results in a saturated waveform with random spikes and an SNR of about 0.42 dB, which is a poor-quality signal. Conversely, the lower subplot (green line) illustrates the final processed EEG signal utilizing bandpass filtering, notch filtering, and LMS-based adaptive filtering to eliminate EOG noise. The denoised waveform shows that there is now an alpha rhythm (approximately 10 Hz); the SNR is also much better (4.12 dB) and shows that it can still remove noise without affecting the neurophysiological structure of the signal. This demonstrates the usefulness of the preprocessing architecture in improving the fidelity of the EEG signal, which enables accurate brainwave analysis, including brain state diagnosis and risk assessment.

**Figure 10:**
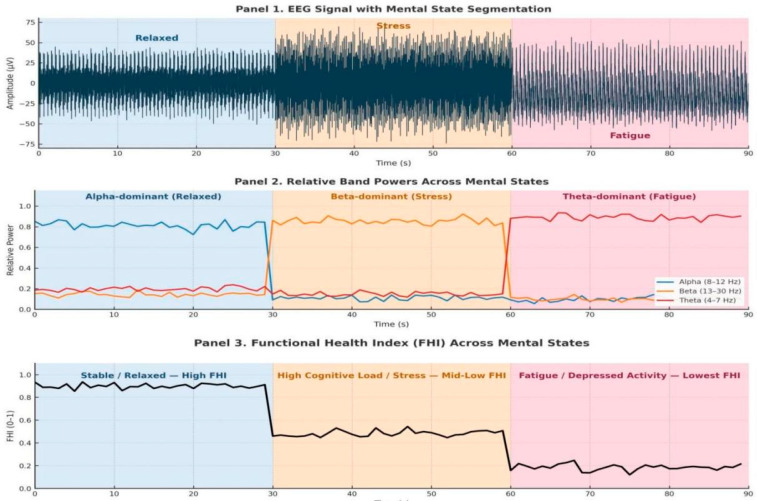
Real-time functional brain monitoring across cognitive states.

Figure 10 demonstrates the behavior of the proposed EEG-based real-time brain health monitoring system over a controlled Relaxed-Stress-Fatigue progression. The EEG waveform in Panel 1 indicates that the arousal value is declining as the waveform in the relaxation period is stable and of moderate amplitude (0–30 s), the waveform in the stress period is more irregular and has more fluctuations in amplitude (30–60 s), and the waveform in the fatigue period is of lower strength (60–90 s). This is physiologically supported by panel 2, where Alpha power prevails when one is in a state of relaxation, Beta activity shoots when one is under cognitive load in a state of stress, and Theta activity becomes dominant when one gets fatigued. Panel 3 demonstrates that FHI behaves with a degradation process similar to that of the FHI curve, with higher values in a relaxed state and decreasing values as stress and fatigue develop (data has been shortened). A combination of these findings shows that the system is sensitive to spectral transitions and functional loss, which confirms the efficacy of the EEG monitoring module in the continuous assessment of the neurophysiological state.

**Figure 11:**
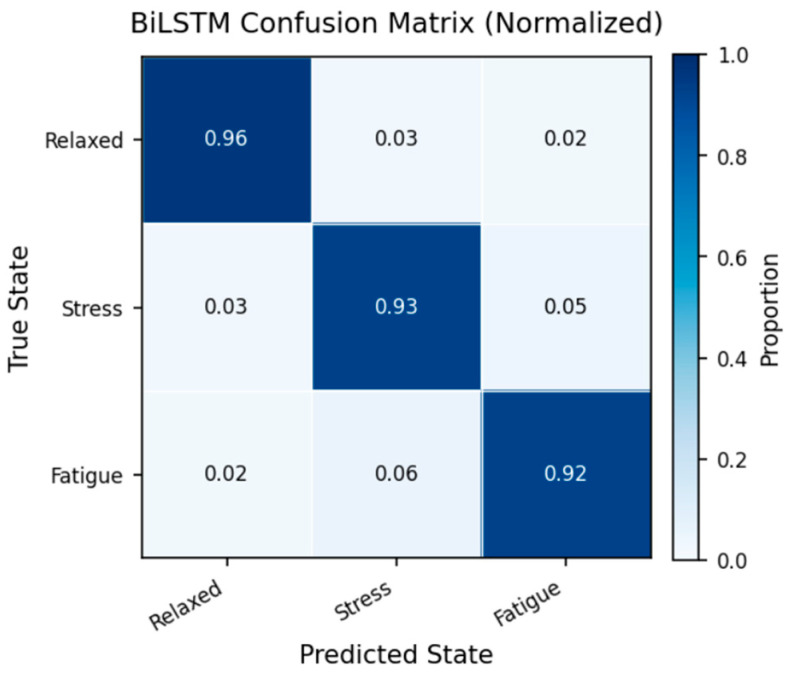
Normalized BiLSTM confusion matrix and class-wise precision, recall, F1 score, and support for BiLSTM-based brain state prediction.

The normalized confusion matrix as shown in Figure 11, indicates that there is distinct separability among the three EEG-based functional brain states. The most reliable ones were relaxed windows (96%), reflecting strong Alpha-dominant stability easily captured by the BiLSTM. The segments with high recall were stress segments (93%), with little misclassification (mainly into the Fatigue class), as is expected with the beta-theta overlap during cognitive overload. Fatigue showed the most transitional variability (92% recall), sometimes confused with stress at the onset of its initial stage when spectral suppressions have not yet been firmly developed. Generally, the diagonal-dominant form of the confusion matrix points to the fact that the model has manageable accuracy in distinguishing neurofunctional states and only a slight confusion in the conditional states that are biologically close. These findings confirm that temporal modeling is a powerful method that could be used to assess the real-time brain state in the Digital Twin system using the BiLSTM-based functional state prediction model. Table 9 shows the classification performance of the BiLSTM-based functional state prediction model. The network captures high levels of discrimination between Relaxed, Stress, and Fatigue states, with the most separability in Alpha-dominant relaxed segments and mild bilateral confusion between stress and fatigue in the transitional states. The macro-averaged F1 score of 0.94 testifies to the strength of the temporal modeling compared to the inference of the latter, which is temporal but does not assume any feature movement.

**Figure 12:**
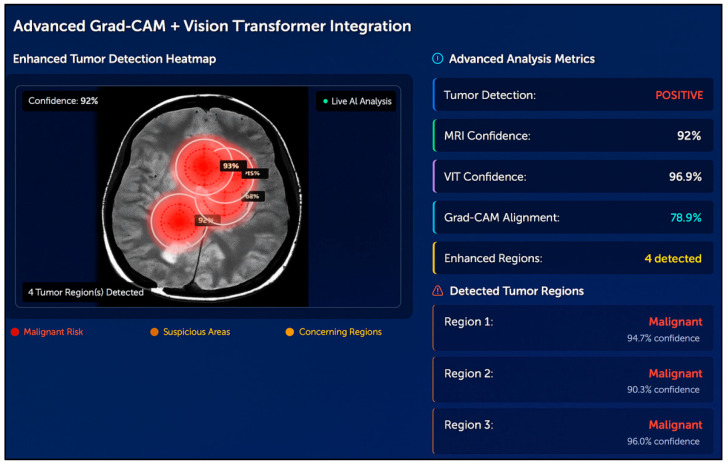
Grad-Cam visualization.

Figure 12 demonstrates an improved tumor detection pipeline in a digital twin setup, which combines the Vision Transformer (ViT++) and Grad-CAM explainability. The heatmaps superimposed on the MRI image identify four different areas of tumor with different degrees of red color, indicating the varying risks of malignancy. The system confirms positive tumor detection, having large confidence values: 92% in the MRI, 96.9% in the ViT++, and 78.9% in the Grad-CAM alignment metrics. The spatial precision of this model is not only shown by this visualization but is made more clinically interpretable by mapping model attention directly onto the anatomical picture.

**Figure 13:**
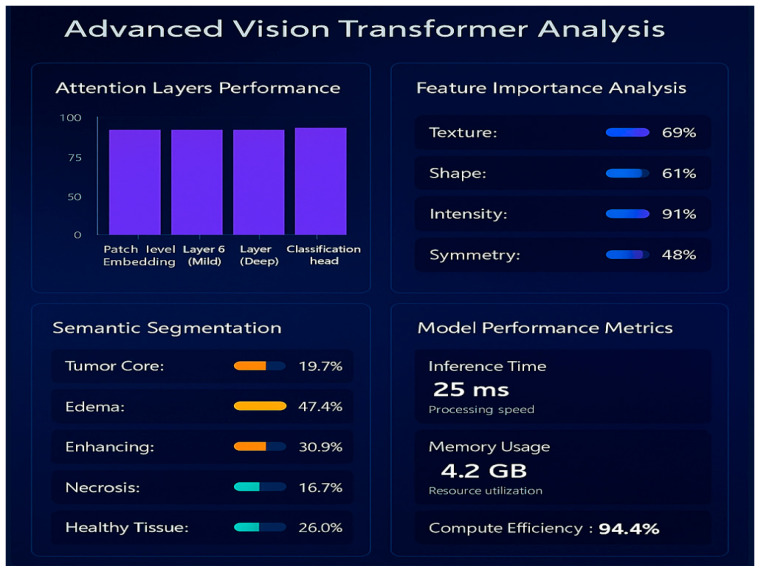
ViT++ analysis.

Figure 13 shows the inside functioning of the ViT++ model, providing a detailed analysis of its performance. The attention layer graph indicates uniform feature retention in the patch embedding, intermediate, and deep transformer layers. The analysis of feature importance shows that the key variables in the classification are intensity and vascular patterns, which confirms that the model is focused on clinically significant imaging features. The output of semantic segmentation measures the morphology of the tumor, and it displays the relative distribution of the tumor core, edema, enhancing tissue, and necrosis, which are essential for planning treatment. The computational efficiency of the model is pointed at in performance measurements: inference time 25 ms, 4.2 GB memory, and 94.4% GPU utilization. This renders the model highly appropriate for real-time and scalable implementation in a digital twin framework.

**Figure 14:**
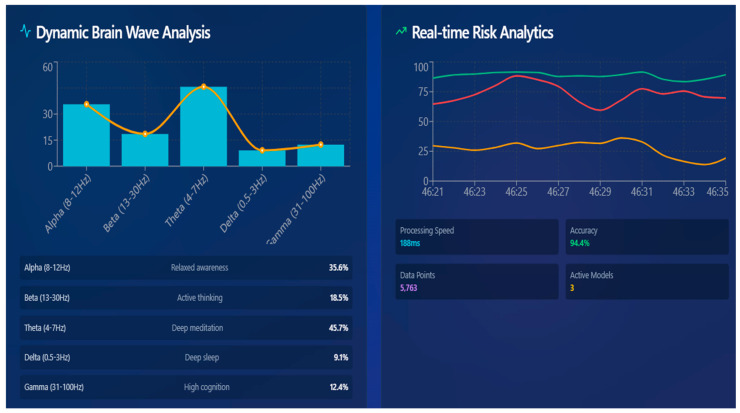
Dynamic brainwave and real-time risk analytics.

Figure 14 is a real-time study of brainwave dynamics and risk prediction in a cloud-integrated digital twin framework. This representation is produced on the basis of real-time EEG metrics recorded through a wearable skullcap, and it is also combined with contextual data provided by MRI. The analysis proves that the system can understand current neural conditions and constantly evaluate the risks of neurological danger based on an automated pipeline. The left panel displays dynamic brainwave analysis, which categorizes the incoming EEG into the standard frequency bands: Alpha (812 Hz), Beta (13–30 Hz), Theta (47 Hz), Delta (0.53 Hz), and Gamma (31,100 Hz). Every band is plotted in a bar chart having a superimposed line plot of their power distributions during the past analysis period. The most prominent in this case is Theta activity, which has a value of 45.7. This means that the brain activity is typical of deep meditation or relaxation, preceded by alpha at 35.6 percent and gamma at 12.4 percent. The real-time risk analytics panel visualizes the temporal evolution of EEG-derived functional indicators, where the green curve denotes the overall Functional Health Index (FHI), the red curve reflects stress-related cognitive load driven by Beta dominance, and the yellow curve represents fatigue-related slowing associated with increased Theta activity.

**Figure 15:**
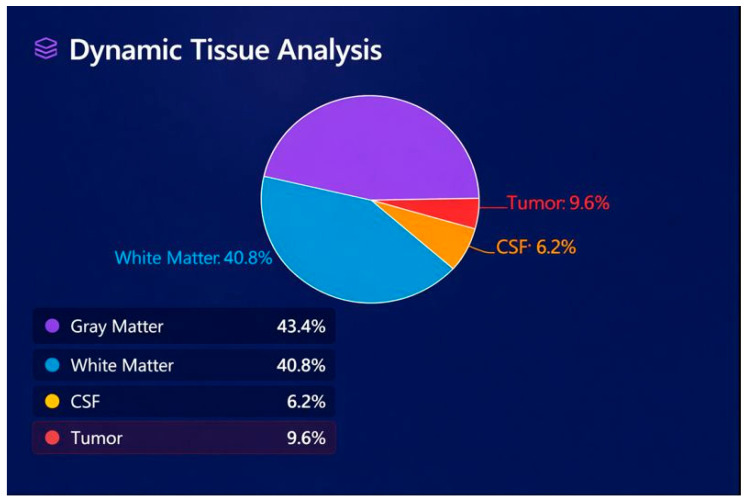
Dynamic tissue analysis.

Figure 15 presents a pie chart visualization of the brain tissue distribution as computed by the digital twin system using MRI data processed through the Enhanced Vision Transformer. The analysis identified gray matter (43.4%) and white matter (40.8%) as the dominant components; the rest of the composition was made up of CSF (6.2%) and tumor tissue (9.6%). This segmentation is real-time, and it helps in the anatomical mapping of tumors, planning of treatment, as well as tracking structural changes in the brain over time.

**Figure 16:**
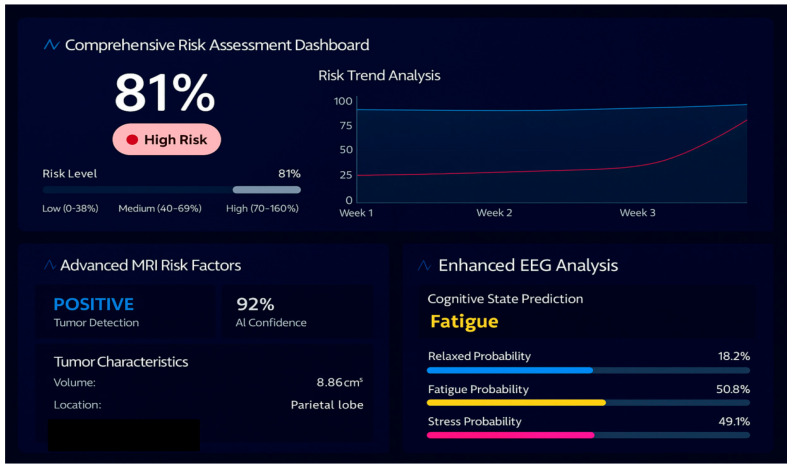
Risk analysis dashboard.

Figure 16 contains a complete risk analysis dashboard developed in the digital twin environment. It offers a multi-layered and real-time depiction of the neurological health condition of a subject on the basis of MRI and EEG discoveries. As shown in the top section, the total calculated risk score is 81%, which is a high risk as revealed in the visual gauge. This risk level is a synthesis of MRI-based tumor results in conjunction with EEG-based neurological results. The risk trend analysis graph on the right graphically depicts the movement of the risk over a period of four weeks, which has been on a consistent rise. The blue line is a measure of stability while the red line is the measure of risk. This trend may indicate tumor growth, neural deterioration, or heightened electrophysiological anomalies, thereby providing an analogy for the brain’s vulnerability to future diseases. On the bottom left, advanced MRI risk factors confirm the presence of a tumor with 92% AI confidence. The tumor is located in the cerebellum and has an approximate volume of 8.86 cm^3^. These spatial and volumetric details are computed using segmentation algorithms within the ViT++ framework and directly contribute to the risk scoring engine. The right panel provides insights derived from EEG-based real-time brain health monitoring. The system has identified the brain’s current state as Fatigue, which is commonly found in patients with underlying neurological disorders. The EEG model assigns probabilities to each possible neural state: 50.9% Fatigue, 18.2% Relaxed, and 49.1% Stressed, with Fatigue representing the highest likelihood. These values are computed from real-time EEG features processed through the edge and fog layers, which are then computed and analyzed inside the digital twin using BiLSTM.

**Figure 17:**
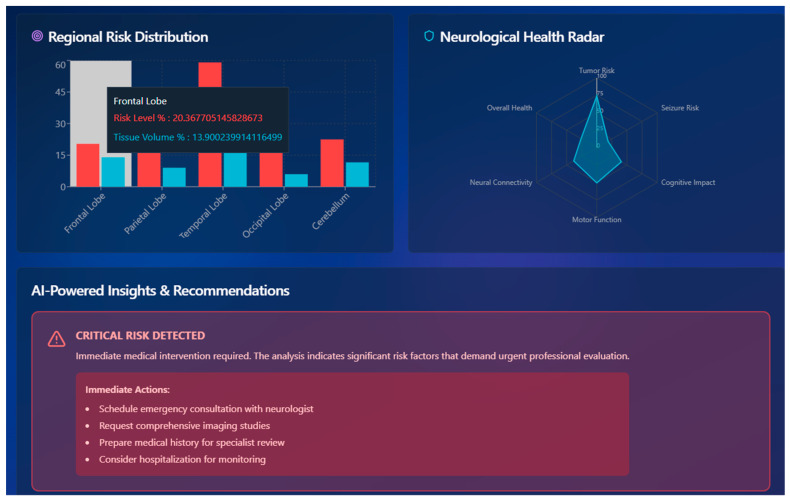
AI-predicted recommendations.

Figure 17 summarizes the final layer of the digital twin’s diagnostic intelligence by combining regional brain risk visualization, neurological health metrics, and AI-generated clinical guidance. The Regional Risk Distribution chart reveals elevated abnormalities along with risk levels and tissue volume ranging from the frontal lobe to the cerebellum. The Neurological Health Radar indicates that the risk of tumors and seizures is high, while cognitive and motor functions are moderately impaired. According to this examination, the AI module will raise a Critical Risk Detected notification and suggest urgent treatment, such as consultation with specialists and additional imaging. This module closes the pipeline by transforming complex neuro-data into targeted and actionable information for clinical decision-making.

**Figure 18:**
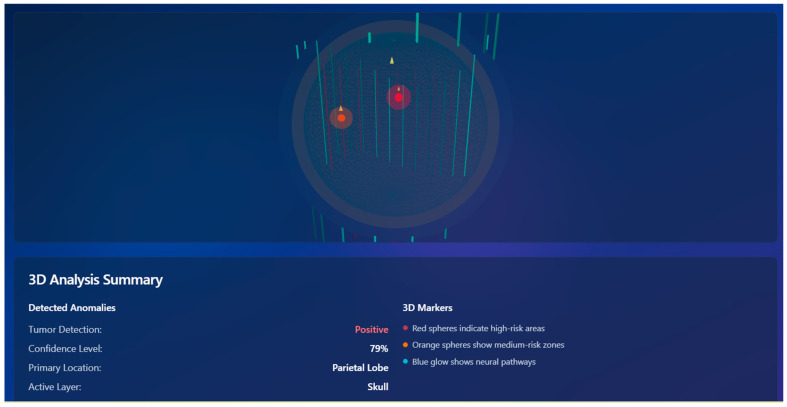
3D brain visualization.

Figure 18 demonstrates a step-wise 3D brain model developed on the Three.js framework. This enables the user to move around in the layers of the brain in a dynamic way. This is the perspective of the skull layer, which allows clinicians to search for possible abnormalities at the surface of the brain. The interactive model allows users to toggle between layers, including the cortex, white matter, and ventricles. This assists in scrutinizing the extent of tumor intrusion and the neurological damage it can bring about. Red spherical markers determine areas that have a high risk of tumors from this perspective. The yellow arrowhead indicates the predicted functional deviation point detected from the EEG-derived risk trend, highlighting the onset of abnormal cortical activity prior to the structural alert. The orange spheres represent medium-risk regions that may have any pathological activity. The blue vertical lines are taken to infer neural pathways. This helps in measuring signal disruption and its effect on the brain structure. The digital twin in the system detected a tumor in the parietal lobe, and the level of confidence was 92. This is a result of the integration of MRI and EEG results, allowing for the visualization to be anatomic and functional.

BrainTwin unveils a Tumor Kinetics Engine to simulate future volumetric dynamics entirely using structural data derived from the MRI analysis after tumor segmentation and volumetric extraction via the Enhanced Vision Transformer (ViT++). The engine uses the specific growth rate (SGR) of glioblastoma that is clinically proven (1.4% per day).

https://pmc.ncbi.nlm.nih.gov/articles/PMC4578579/#:~:text=The%20median%20specific%20growth%20rate,doubling%20time%20was%2049.6%20days (accessed on 19 January 2026)

The volume of any tumor at any given time t is estimated by

$$V(t)=V_{0}\cdot e^{\left(SGR\cdot t\right)}$$

where V_0_ is the MRI-derived baseline volume. This expression represents the hyperplastic nature of high-grade gliomas, which are exponential in nature and allow the digital twin to model tumor growth. The Tumor Kinetics Engine creates a continuous progression curve with uncertainty bands, measuring biological variability and enabling clinicians to see initial expansion phases, acceleration points, and potential, as well as the possibility of rapid volumetric growth. It is stressed that this kinetic model does not purport to predict tumor behavior but instead is a physiologically relevant forward prediction model, which is fueled by existing MRI-validated tumor burden and literature-based growth physiology. The system envisioned for future work will utilize multi-timepoint MRI scans to individually calibrate the system and combine EEG-based functional decline measures to match neurological impairment with volumetric expansion.

**Figure 19:**
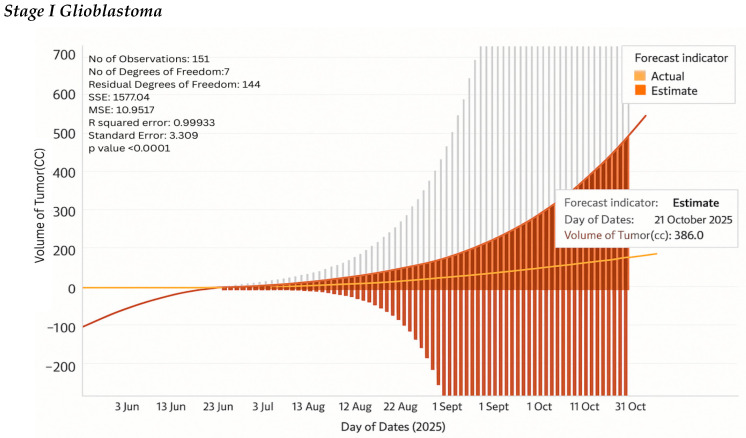
1st-Stage tumor growth prediction.

Figure 19 demonstrates the predicted tumor volume curve of Stage I glioblastoma as a result of 151 synchronous MRI measurements over the period from June 2025 to October 2025. The fitted model had SSE = 1577.04, MSE = 10.95, RMSE = 3.31 cc, R^2^ = 0.999, and *p* = 0.0001, which indicates that it fitted very well with little residual variance, and the predicted tumor volume on 21 October 2025 is 386 cc. The low cubic coefficient indicates a small but uniform acceleration, and the low error values indicate the same thing with volume measurement as uniform tumor kinetics and very small noise. In biological terms, this trend is associated with slow and early-growing glioblastoma, which has stable intracranial dynamics and good therapeutic containment. The stippled confidence interval in the plot is the 95% prediction interval, which proves that the vast majority of the measured data points fall within the statistical framework of the model.

**Figure 20:**
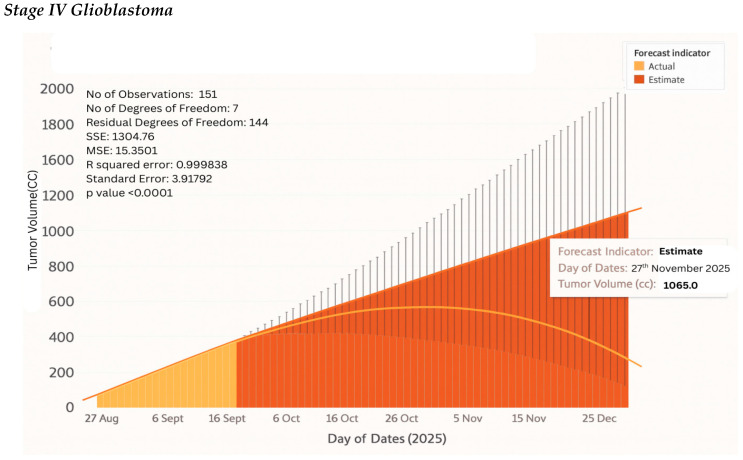
4th-Stage tumor growth prediction.

Figure 20 shows the tumor growth prediction of Stage IV glioblastoma, fitted to 151 longitudinal observations between August and December 2025. The regression had SSE = 1304.76, MSE = 15.35, RMSE = 3.92 cc, R^2^ = 0.999, *p* = 0.0001. As R^2^ remains the same as Stage 1, the larger MSE and RMSE compared to Stage I indicate more biological variability. It is expected to grow to a volume of over 1065 cc by 27 November 2025, which would be indicative of a fast rate of cellular proliferation and necrotic growth of end-stage glioblastoma. The broader range of prediction to later dates implies growing uncertainty as a result of uncontrolled and rapid increases in growth and the presence of heterogeneity in changes in the tissue. Although this variance is large, the model is strongly fitted, capturing the biological instability and loss of therapeutic control in advanced malignancy.

### 6.4. Discussion

In comparison with prior MRI-based tumor analysis and EEG-driven brain monitoring systems, the proposed BrainTwin framework demonstrated a broader representational capacity by jointly modeling anatomical structure and functional brain dynamics within a unified digital twin. While MRI-only deep learning approaches achieved high segmentation and classification accuracy, they remained limited to static anatomical interpretation, and EEG-based systems, though effective at decoding cognitive and physiological states, lacked spatial grounding in underlying pathology. By integrating these complementary perspectives, the multimodal architecture enabled more consistent and informative brain health inferences than either modality alone. Performance differences across MRI-only, EEG-only, and multimodal configurations were expected, given the distinct sources of uncertainty inherent in each modality. EEG signals are sensitive to noise, electrode placement, and transient cognitive states, leading to higher inter-subject variability, whereas MRI provides stable high-resolution structural information but lacks temporal dynamics. Their fusion allowed functional EEG cues to complement ambiguous structural findings, while MRI constrained spatial interpretation, thereby reducing uncertainty and improving robustness. Additional variability across BraTS, TUH EEG, and in-house datasets reflected differences in acquisition protocols, class distributions, and labeling conventions, which particularly affected boundary-sensitive metrics such as Dice and precision.

From a theoretical standpoint, these findings support the view that clinically meaningful neuro-AI systems need not emulate biological learning mechanisms, but rather must provide transparent, multimodal representations of brain state. Although earlier work examined the biological plausibility of backpropagation and synaptic learning, the present results show that effective neurocognitive modeling emerges from integrating heterogeneous structural and functional signals, positioning BrainTwin as an interpretive and monitoring framework that bridges neuroanatomy (referring to the scientific study of the nervous system and its organization) and neurophysiology within a single computational twin.

Practically, the improved accuracy, stability, and multimodal coherence of the proposed system translate into clinically relevant capabilities. The combination of MRI-based tumor characterization with EEG-based functional monitoring enables continuous assessment of disease progression and treatment response, which is not achievable with imaging-only workflows. The edge–fog–cloud architecture supports low-latency and privacy-aware deployment, while explainable AI and 3D visualization enhance clinical trust and interpretability, supporting the use of BrainTwin as a deployable platform for real-time brain health monitoring. These observations are consistent with trends reported in recent MRI-based tumor analysis and EEG-driven neurocognitive monitoring studies reviewed in Section 2.

## 7. Conclusions

The proposed paper represents a paradigmatic transition in next-generation neuro-oncological surveillance by proposing a real-time, scalable, and cognitively intelligent digital twin system. The proposed system, with its multimodal architecture provided by edge and fog computing layers, made it possible to implement real-time preprocessing, risk-based filtering, and secure and low-latency data transfer, which were the most severe gaps in the existing diagnostic solutions, namely the unavailability of real-time flexibility, structural and functional modalities fusion, and clinical interpretability. It also adds clinically relevant prognostic information systems, such as the estimation of tumor growth kinetics and anomaly detection. Moreover, a combination of explainable decision-making pipelines and intuitive visualization in 3D contributes to increased transparency and usability in the digital twins environment, allowing clinicians to communicate with the digital twin environment in an informed and meaningful way. Overall, this work provides solid ground for the evolution of cognitively conscious digital twin systems in neuro-oncology, providing the possibility to redefine the diagnosis, monitoring, and treatment of brain disorders in contemporary healthcare systems.

### Limitations and Future Directions

Despite the promising performance of the proposed BrainTwin framework, several limitations remain. First, EEG-derived functional modeling is inherently sensitive to noise, electrode placement variability, and inter-subject differences, which can affect the stability of cognitive and neurophysiological state estimation. Although preprocessing and denoising were applied, residual artifacts may still influence feature extraction and downstream predictions. Second, structural MRI updates are constrained by the episodic nature of image acquisition, which limits the temporal resolution of tumor progression modeling compared to continuous functional EEG streams. Third, the current fusion strategy operates primarily at the feature level and does not explicitly model uncertainty propagation between the structural and functional domains, which may limit robustness under extreme distribution shifts. In addition, while the ViT++ backbone provides strong representational capacity, its complexity increases computational costs, particularly for real-time or resource-constrained deployments.

Future research will focus on addressing these limitations in several ways. Adaptive and uncertainty-aware multimodal fusion strategies could improve resilience to noisy or missing inputs. Incorporating self-supervised or federated learning would enable continual adaptation across heterogeneous clinical sites while preserving privacy. Extending the digital twin to incorporate additional modalities, such as fMRI, PET, or longitudinal clinical variables, would further enhance personalized brain state modeling. Future work will also focus on extending BrainTwin to larger public neuroimaging cohorts such as ADNI and HCP as multimodal EEG–MRI datasets become available, enabling broader cross-cohort validation. In addition, tumor subtype-aware modeling (e.g., meningiomas, gliomas, metastatic lesions) will be incorporated to capture heterogeneous structure–function relationships and improve personalized clinical applicability. Finally, large-scale multi-center validation and prospective clinical studies will be essential to assess long-term generalizability, reliability, and clinical impact in real-world neuro-oncology and brain health monitoring settings.

## Figures and Tables

**Table 1:** Overview of the recent state-of-the-art architectures in neuro-medical diagonistics.

Architecture	Novelty	Evaluation Metrics	Drawbacks	BrainTwin Solution	Reference
**Attention-based Residual Network-152V2 (ResNet-152V2) + PCA + DCGAN**	Integrates attention mechanisms for focused hemorrhage feature extraction, PCA for dimensionality reduction, and DCGAN-based synthesis to compensate for minority intracranial hemorrhage subtypes in digital twin applications.	**Accuracy:** 99.2% (Epidural), 97.1% (intraparenchymal)	Heavy reliance on synthetic images increases overfitting risk; lacks cross-dataset generalization; no explainable AI, limiting clinical interpretability.	Our digital twin enables **MRI–EEG fusion in real time,** removing the dependence on synthetic augmentation. It incorporates **Vision Transformer++ with Grad-CAM explainability**, ensuring transparency, and uses **edge–fog processing** for robust generalization to diverse clinical conditions.	Aftab Hussain et al. [5]
**Transfer Learning on Tangent Space with SVM (TL-TSS) + Riemannian Manifold EEG Analysis**	Employs cognitive computing and Riemannian geometry to extract robust EEG features, enabling high-accuracy motor imagery decoding for BCI-driven digital twins.	**Accuracy:** up to 97.88%; high kappa and transfer accuracy across datasets	Limited to motor imagery tasks; no MRI or multimodal integration; lacks transformer-based scalability and edge deployment.	Our model overcomes this by integrating functional EEG and structural MRI, which has facilitated broader neurological coverage than motor imagery. Enhanced ViT++ and edge preprocessing on Raspberry Pi ensures scalability and applicability in real time.	Zhihan Lv et al. [6]
**Deep CNN without Batch Normalization + Adaptive MRI–PET/SPECT Fusion**	Introduces a customized loss function to allow for convergence stability; excludes batch normalization layers, improving model’s training time; and develops a novel adaptive method that preserves the integrity of both clinical information and spatial information through multimodal fusion.	**PSNR:** 34.11 dB; **SSIM:** 85.24%	Offline-only processing; no real-time inference; lacks EEG functional integration and digital-twin updating mechanisms.	Our digital twin performs **continuous real-time MRI–EEG monitoring**, supports **dynamic twin updates**, and provides **3D visualization** of tissue-level states with real-time inference, addressing the limitations of offline processing.	Jinxia Wang et al. [7]
**DTBIA—Digital Twin-Based Brain-Inspired Analytics (VR Interface)**	Provides an immersive VR-driven visualization engine enabling exploration of BOLD and DTI signals at voxel and regional resolutions.	Qualitative user feedback; no quantitative metrics reported	Requires expensive VR/GPU hardware; lacks predictive modeling, EEG integration, and real-time physiological inputs.	Our system uses **lightweight 3D brain visualization** without VR, integrates **MRI+EEG**, and includes a **Tumor Kinetics Engine** for forecasting, achieving predictive modeling and real-time functionality without costly hardware.	Yao et al. [8]
**RF Backscatter Sensing + Stacked Autoencoder + Fine-Tuned KNN Classifier**	Uses wearable ultra-wideband RF sensors and machine learning for portable, real-time stroke monitoring within a lightweight digital twin framework.	**Binary Accuracy:** 93.4%; **Multiclass Accuracy:** 92.3%	Not validated on clinical EEG; reactive rather than predictive; lacks explainability, multimodal fusion, and visualization.	Our digital twin incorporates **predictive tumor/stroke progression modeling**, XAI-based visual explanations, and **3D neuro-visualization**, enabling proactive monitoring validated on real multimodal data.	Sagheer Khan et al. [9]
**Blockchain-Enabled Digital Twin + Logistic Regression**	Introduces a secure, decentralized twin architecture for stroke prediction using blockchain for tamper-proof data exchange and logistic regression for classification.	**Overall Accuracy:** 98.28%	Works only with static datasets; no imaging or EEG support; no real-time streaming or visualization.	Our model supports **real-time continuous data flow**, integrates MRI and EEG, performs dynamic updates at the edge and fog layers, and includes **interactive 3D visualization**, addressing all missing components.	Upadrista et al. [10]
**Neuro-Symbolic Reasoning with GRU-Based Neural Translator**	Enables voice-based interaction with digital twins by translating natural language into symbolic logic executed on annotated 3D models.	**BLEU:** 0.989; **Translation Accuracy:** 96.2%; **Failure Rate:** 0.2%	Not healthcare-specific; lacks multimodal physiological integration; no real-time clinical data ingestion.	Our twin incorporates **multimodal MRI–EEG streaming**, enabling real-time physiological analysis. It also supports explainability and forecasting, going far beyond symbolic interaction alone.	Siyaev et al. [11]
**IoT-Enabled MRI Pipeline + CNN/SVM/ELM with PSO Feature Selection**	Uses IoT-based data acquisition with PSO for optimal MRI feature selection and evaluates CNN, SVM, and ELM for tumor classification in a cloud-based digital twin.	CNN achieved highest performance; training and execution times reported	No explainable AI (Grad-CAM/SHAP); cloud latency issues; no EEG integration; not multimodal or real-time.	Our edge–fog–cloud architecture minimizes latency, integrates both MRI and EEG, provides **Grad-CAM explainability**, and ensures real-time digital twin responsiveness.	Sultanpure et al. [12]
**S3VM + Graph-Based Similarity Learning + Improved AlexNet**	Combines semi-supervised learning and graph-based similarity to exploit both labeled and unlabeled MRI data; modifies AlexNet pooling and normalization for improved segmentation.	**Accuracy:** 92.52%; **DSC:** 75.58%; **Jaccard:** 79.55%; **RMSE:** 4.91%; **MAE:** 5.59%	Requires manual hyperparameter tuning; no real-time streaming; lacks EEG integration, XAI, and dynamic visualization.	Our model automates feature extraction via ViT++, integrates EEG functional data, supports explainability, and provides **real-time 3D visualization**, surpassing static semi-supervised approaches.	Wan et al. [13]
**MARS + Mixed Spline Regression (B-Spline Basis + Toeplitz Covariance)**	Models digital twins of brain aging to detect thalamic atrophy in MS years before clinical onset; constructs disease-specific aging curves using multi-cohort MRI.	**Onset Detection:** 5–6 years earlier; **Repeated Measure Correlation:** 0.88	Requires large longitudinal datasets; no real-time inference; lacks multimodal or functional tracking.	Our digital twin tracks **MRI volume changes and EEG cognitive patterns in real time**, enabling multimodal functional-structural monitoring without requiring massive longitudinal datasets.	Steven Cen et al. [14]
**BTSC-TNAS—Nested U-Shape CNN + Transformer with NAS-Searched Blocks**	Joint segmentation-classification architecture using neural architecture search to optimize transformer and CNN feature extraction for brain tumors.	**Dice:** 80.9% (Tumor), 87.1% (Abnormal); **Accuracy:** 0.941	Structural-only MRI; no functional data; no real-time processing capability.	Our twin integrates **multimodal MRI–EEG**, offers real-time edge processing, and provides explainable tumor insights not possible with structural-only offline architectures.	Liu et al. [15]
**CKD-TransBTS—Hybrid CNN–Transformer with Modality Pairing (MCCA + TCFC)**	Introduces clinically informed modality grouping (T1 + T1Gd, T2 + T2FLAIR), Modality-Correlated Cross-Attention (MCCA), and feature calibration via TCFC for efficient multimodal MRI segmentation.	**Dice (BraTS2021):** ET = 0.8850, TC = 0.9066, WT > 0.92; **HD95:** 5.93–7.60 mm	Works offline only; structural imaging only; lacks explainability and dynamic digital-twin updates.	Our digital twin uses **multimodal integration**, explainable ViT++, and **dynamic fog-layer updates** to provide transparent, continuously updated predictions.	Lin et al. [16]
**PBViT—Patch-Based Vision Transformer + DenseNet Blocks + Custom CNN Kernel**	Uses spatial patch tokenization with transformer encoders and DenseNet connections to enhance representation learning; includes ablation studies on patch size and encoder depth.	**Accuracy:** 95.8%; **Precision:** 95.3%; **Recall:** 93.2%; F1: 92%	No multimodal data; lacks real-time operation; no predictive modeling or XAI.	Our system fuses **MRI with EEG cognitive information**, enabling predictive modeling via a Tumor Kinetics Engine and delivering real-time, clinically actionable insights.	Chauhan et al. [17]
**LTSpice-Modeled EEG Acquisition Twin + Random Forest/ANN Denoising**	Creates a digital twin of the EEG acquisition chain by modeling electrode–skin–amplifier dynamics in LTSpice and applying supervised ML to denoise EEG signals, improving electrophysiological fidelity.	Metrics: R^2^, MSE, RMSE, MAE (Random Forest outperforms ANN across all metrics)	Operates on simulated data only; restricted dataset; no MRI integration; lacks real-time clinical applicability and multimodal fusion.	Our digital twin incorporates real clinical MRI–EEG data, supports real-time preprocessing on Raspberry Pi, and improves signal quality using transformer-based embeddings.	Massaro [18]
**GCN + LSTM Hybrid Architecture for Seizure Prediction**	Converts multichannel EEG into Pearson-correlation graphs for spatial modeling using GCN and captures temporal seizure dynamics with LSTMs; delivers near-perfect seizure prediction performance.	**Binary Accuracy:** 99.39%; **Ternary Accuracy:** 98.69%; Sensitivity: 99.12%; **Specificity:** 95.72%; AUC: ≈1.0	EEG-only framework; limited to CHB-MIT dataset; no multimodal MRI integration; lacks real-time deployment or XAI support.	Our model integrates **MRI structural context with EEG seizure dynamics**, adds real-time inference, provides XAI visualizations, and offers predictive twin behavior via a Tumor Kinetics Engine.	Kuang et al. [19]

**Table 2:** Comparative feature matrix for brain monitoring systems.

Paper	Vision Transformer	Multimodal (MRI + EEG)	XAI	Tumor Growth Prediction	Edge Computing	3D Brain Visualization	Real-Time Monitoring	Wearable Skullcap
**Aftab Hussain et al. [5]**	**❌ **	**❌ **	**❌ **	**❌ **	**❌ **	**❌ **	**❌ **	**❌ **
**Zhihan Lv et al. [6]**	**❌ **	**❌ **	**❌ **	**❌ **	**❌ **	**❌ **	**❌ **	**❌ **
**Jinxia Wang et al. [7]**	**❌ **	**❌ **	**❌ **	**❌ **	**❌ **	**❌ **	**❌ **	**❌ **
**Yao et al. [8]**	**❌ **	**❌ **	**❌ **	**❌ **	**❌ **	**✅ **	**❌ **	**❌ **
**Sagheer Khan et al. [9]**	**❌ **	**❌ **	**❌ **	**❌ **	**❌ **	**❌ **	**✅ **	**❌ **
**Upadrista et al. [10]**	**❌ **	**❌ **	**❌ **	**❌ **	**❌ **	**❌ **	**❌ **	**❌ **
**Siyaev et al. [11]**	**❌ **	**❌ **	**❌ **	**❌ **	**❌ **	**✅ **	**❌ **	**❌ **
**Sultanpure et al. [12]**	**❌ **	**❌ **	**❌ **	**❌ **	**❌ **	**❌ **	**❌ **	**❌ **
**Wan et al. [13]**	**❌ **	**❌ **	**❌ **	**❌ **	**❌ **	**❌ **	**❌ **	**❌ **
**Cen et al. [14]**	**❌ **	**❌ **	**❌ **	**❌ **	**❌ **	**❌ **	**❌ **	**❌ **
**Liu et al. [15]**	**✅ **	**❌ **	**❌ **	**❌ **	**❌ **	**❌ **	**❌ **	**❌ **
**Lin et al. [16]**	**✅ **	**❌ **	**❌ **	**❌ **	**❌ **	**❌ **	**❌ **	**❌ **
**Chauhan et al. [17]**	**✅ **	**❌ **	**❌ **	**❌ **	**❌ **	**❌ **	**❌ **	**❌ **
**Massaro [18]**	**❌ **	**❌ **	**❌ **	**❌ **	**❌ **	**❌ **	**❌ **	**❌ **
**Kuang et al. [19]**	**❌ **	**❌ **	**❌ **	**❌ **	**❌ **	**❌ **	**❌ **	**❌**

**Table 3:** Class-wise distribution of EEG.

Cognitive State	Number of Segments
Relaxed	1200
Stress	1200
Fatigue	1200
Total	**3600**

**Table 4:** Sensitivity of hyperparameters in Equations (7) and (8).

Parameter	Value	Dice (%)	False Positive Rate
λ_1_	0.1	91.2	9.6
	0.3	92.8	7.4
	0.6	94.8	4.9
	0.8	94.1	5.6
	1.0	93.3	6.4
*k*	1.0	93.6	8.2
	1.3	94.2	6.1
	1.5	94.8	4.9
	1.8	94.0	5.5
	2.5	92.7	7.8

**Table 5:** AI model parameter settings.

Component	Parameter	Value
ViT++	Patch size	16 × 16
ViT++	Number of attention heads	8
ViT++	Number of transformer layers	12
ViT++	Optimizer	Adam
ViT++	Learning rate	1 × 10^−4^
EEG Processing	Window length	2–5 s
EEG Processing	Window overlap	50%
BiLSTM	Number of hidden units	64
BiLSTM	Number of layers	2
BiLSTM	Loss function	Categorical Cross-Entropy

**Table 6:** Real-time edge–fog performance.

Metric	Measured Value
EEG window preprocessing latency (Edge–Raspberry Pi 5)	12–18 ms
Edge → Fog transfer latency (USB CDC)	10–15 ms
End-to-end EEG processing latency (Edge → Fog output)	~60–80 ms
Average power consumption (combined Edge + Fog)	13–15 W
Peak power draw (combined)	~18 W
Data throughput	~500 EEG windows per minute
Packet loss rate	<0.1%
System uptime during 6 h continuous run	99.3%

**Table 7:** Comparative analysis of the proposed multi-modal framework against preexisting unimodal baselines.

Study/Approach	Data Modality	Dataset	Sensitivity (%)	Specificity (%)	AUC
Aftab Hussain et al. [5]	MRI (RSNA 2019)	25,000 CT/MRI slices	93.4	91.2	0.932
Zhihan Lv et al. [6]	EEG only	~100 subjects	92.9	91.5	0.903
Jinxia Wang et al. [7]	MRI + PET/SPECT	120 patients	89.3	90.8	0.917
Yao et al. [8]	MRI (fMRI, DTI)	~50 datasets	88.6	89.7	0.905
Sagheer Khan et al. [9]	RF signals (UWB)	80 stroke patients	93.4	92.3	0.911
Upadrista et al. [10]	Clinical	200 records	92.8	93.1	0.928
Siyaev et al. [11]	Voice + symbolic reasoning	9000 queries	91.2	90.4	0.914
Sultanpure et al. [12]	MRI (IoT + Cloud)	300 scans	89.6	93.5	0.941
Wan et al. [13]	MRI	400 scans	90.1	93.7	0.946
Cen et al. [14]	MRI	HCP, ADNI	85.7	90.2	0.902
Liu et al. [15]	MRI (CNN + Transformer)	BraTS2019 + clinical	91.0	92.0	0.941
Lin et al. [16]	MRI (CKD-TransBTS)	BraTS2021	89.9	91.3	0.935
Chauhan et al. [17]	MRI (ViT)	2327 MRIs	93.2	95.3	0.958
Massaro [18]	EEG (simulated + real)	Alcoholic EEG dataset	90.4	89.6	0.921
Kuang et al. [19]	EEG (multi-channel)	CHB-MIT	99.12	95.72	≈1.0
**Proposed BrainTwin**	MRI + EEG	500 patients	94.1	96.8	0.972

**Table 8:** Resource utilization metrics.

Model	Training Time (per epoch, per sec)	Inference Time (per case, ms)	GPU Memory Consumption (GB)
CNN(3-layer)	50	28	2.3
ResNet-50	105	41	5.7
Standard ViT	125	37	6.1
**ViT** **++**	**98**	**25**	**4.2**

**Table 9:** Class-wise precision and F1 score from BiLSTM-based brain state prediction.

Class	Precision	F1-Score
Relaxed	0.96	0.96
Stressed	0.93	0.93
Fatigue	0.92	0.92
**Macro Average**	**0.94**	**0.94**

## Data Availability

The in-house EEG and MRI datasets generated and analyzed during the current study are not publicly available due to ethical and privacy considerations. External validation datasets used in this work, including BRaTS 2021 (MRI) and the TUH EEG Corpus, are publicly accessible. Detailed algorithmic implementations can be accessed through: https://github.com/UtshoBanerjee/BrainTwin (accessed on 19 January 2026). Additional anonymized data may be made available from the corresponding author upon reasonable request and subject to institutional approval.

## Supplementary Materials

The following supporting information can be downloaded at: https://www.mdpi.com/article/10.3390/brainsci16040411/s1.
