# Supplementary material for: BrainTwin-AI: A Multimodal MRI-EEG-Based Cognitive Digital Twin for Real-Time Brain Health Intelligence
Source: Brain Sci. 2026 Apr 13;16(4):411. doi: 10.3390/brainsci16040411 (PMC13114914; doi:10.3390/brainsci16040411)
Supplement: Supplementary file 1 [file brainsci-16-00411-s001.zip › brainsci-4079116-supplementary.pdf]

REFERENTIAL CODE SNIPPETS FOR DETAILED WORKFLOW INTERPRETATION

1) Edge Computing

Algorithm 1: EEG Signal Preprocessing and Feature Extraction at the Edge Layer

Hyperparameters: fs = 500 Hz, BP = [0.5, 45] Hz, f_notch = 50 Hz, L = 2s, μ = LMS step-size
Input: Raw EEG signals E_raw = {e1(t), e2(t), …, en(t)}, n = 8
Output: Feature-enriched EEG packet E_features

```
 procedure EEG-Preprocessing(E_raw)
   # Signal Acquisition
   1:  E_raw ← AcquireSignals(n = 8, fs)
   2:  E_raw ← TimeSync(E_raw)
   # Bandpass Filtering
   3:  for each ei(t) ∈ E_raw do
   4:      ei_bp(t) ← Butterworth(ei(t), BP)
   # Powerline Interference Suppression
   5:  for each ei_bp(t) do
   6:      ei_notch(t) ← NotchFilter(ei_bp(t), f_notch)
   # Adaptive Artifact Removal (LMS)
   7:  for each ei_notch(t) do
   8:      ei_clean(t) ← LMS(ei_notch(t), EOG_ref, μ)
   # Segmentation & Feature Extraction
   9:  windows ← Segment(ei_clean(t), L)
   10: for each w ∈ windows do
   11:     f_w ← {PSD(w), BandPowers, Hjorth, RMS, Entropy}
   12: E_features ← Concatenate(f_w)
   # Packet Preparation
   13: packet ← {Patient_ID, Session_ID, Timestamp, E_features}
   14: packet_enc ← Encrypt_AES256(packet)

   return packet_enc
end procedure
```

2) Fog Computing

Algorithm 2: EEG Risk Evaluation and Secure Forwarding at the Fog Layer

Hyperparameters: θ = 0.75, Classifier = Logistic Regression / Quantized DNN (<100k params)
Input: Feature-enriched packet E_features (from Edge Layer)
Output: Risk-evaluated EEG packet transmitted to Cloud Layer

```
procedure RiskEvaluation(E_features)
   # Packet Reception & Validation
   1:  packet ← Decrypt(packet_enc)
   2:  ValidateSchema(packet)
   3:  Authenticate(packet, HMAC_SHA256, Δt ≤ 5s)
     # Lightweight Risk Scoring
   4:  R ← softmax(W · E_features + b)
   5:  R ← Normalize(R, [0,1]
      # Threshold-Based Filtering
   6:  if R ≥ θ then
```

```
 7: MarkClinical(packet, R)
8:  ForwardToCloud(packet, R)
   else
   9: Cache(packet)
     # Compression & Secure Transmission
 10: payload ← Compress({Patient_ID, Session_ID, Timestamp, R, E_features})
 11: PublishMQTT(payload, QoS = 2, TLS1.3)
       # Fault Tolerance
12: if ACK_received = True then
13:     Log(success)
   else
   14:     Retry(max = 3) or StoreLocally()
   end procedure
```

3) Enhanced Vision Transfromer (ViT++)

```
import torch
import torch.nn as nn
import torch.nn.functional as F

# Patch-Level Attention Diversity (PLAD)
def plad_loss(attn_matrix, coef=1e-3):
    avg_attn = attn_matrix.mean(dim=1)
    entropy = -(avg_attn * torch.log(avg_attn + 1e-8)).sum(-1).mean()
    return -coef * entropy

# Dynamic Thresholding
def dynamic_threshold(logits, q=0.6):
    probs = F.softmax(logits, dim=1)[:, 1]
    cutoff = torch.quantile(probs.detach(), q)
    return (probs >= cutoff).int(), cutoff

# Vision Transformer Variant
class CustomViT(nn.Module):
    def __init__(self, img_dim=224, patch_dim=16, embed_size=128, depth=4, n_heads=4, n_classes=2):
        super().__init__()
        self.patch_proj = nn.Conv2d(1, embed_size, kernel_size=patch_dim, stride=patch_dim)
        num_patches = (img_dim // patch_dim) ** 2
        self.pos_tokens = nn.Parameter(torch.randn(1, num_patches, embed_size))
        self.encoders = nn.ModuleList([
            nn.TransformerEncoderLayer(d_model=embed_size, nhead=n_heads, batch_first=True)
            for _ in range(depth)
        ])
        self.norm = nn.LayerNorm(embed_size)
        self.fc = nn.Linear(embed_size, n_classes)

    def forward(self, x):
        x = self.patch_proj(x).flatten(2).transpose(1, 2)
        x = x + self.pos_tokens
        for layer in self.encoders:
            x = layer(x)
        x = self.norm(x).mean(dim=1)
        return self.fc(x), []
```

4) EEG BiLSTM(Brain State Classifier)

```
import torch
import torch.nn as nn

class BiLSTMClassifier(nn.Module):
    def __init__(self, feat_dim=32, hidden_size=64, layers=2, classes=3):
        super().__init__()
        self.lstm = nn.LSTM(
            input_size=feat_dim,
            hidden_size=hidden_size,
            num_layers=layers,
            batch_first=True,
            bidirectional=True
        )
        self.output = nn.Linear(hidden_size * 2, classes)
    def forward(self, x):
        h, _ = self.lstm(x)   # [B, T, 2*hidden]
        return self.output(h[:, -1, :])
```

5) Multi-Modal Fusion

```
class FusionModel(nn.Module):
    def __init__(self, vit, bilstm, fusion_dim=128, num_classes=2):
        super().__init__()
        self.vit, self.bilstm = vit, bilstm
        self.fc1 = nn.Linear(vit.head.out_features + bilstm.fc.out_features, fusion_dim)
        self.fc2 = nn.Linear(fusion_dim, num_classes)

    def forward(self, mri, eeg):
        mri_out, _ = self.vit(mri)
        eeg_out = self.bilstm(eeg)
        x = torch.cat([mri_out, eeg_out], dim=1)
        return self.fc2(F.relu(self.fc1(x)))
```
